# The architecture of the simian varicella virus transcriptome

**DOI:** 10.1371/journal.ppat.1010084

**Published:** 2021-11-22

**Authors:** Shirley E. Braspenning, Georges M. G. M. Verjans, Tamana Mehraban, Ilhem Messaoudi, Daniel P. Depledge, Werner J. D. Ouwendijk

**Affiliations:** 1 Department of Viroscience, Erasmus MC, Rotterdam, the Netherlands; 2 Department of Molecular Biology and Biochemistry, University of California Irvine, Irvine, California, United States of America; 3 Department of Microbiology, New York University School of Medicine, New York, New York, United States of America; 4 Institute of Virology, Hannover Medical School, Hannover, Germany; University of Florida, UNITED STATES

## Abstract

Primary infection with varicella-zoster virus (VZV) causes varicella and the establishment of lifelong latency in sensory ganglion neurons. In one-third of infected individuals VZV reactivates from latency to cause herpes zoster, often complicated by difficult-to-treat chronic pain. Experimental infection of non-human primates with simian varicella virus (SVV) recapitulates most features of human VZV disease, thereby providing the opportunity to study the pathogenesis of varicella and herpes zoster *in vivo*. However, compared to VZV, the transcriptome and the full coding potential of SVV remains incompletely understood. Here, we performed nanopore direct RNA sequencing to annotate the SVV transcriptome in lytically SVV-infected African green monkey (AGM) and rhesus macaque (RM) kidney epithelial cells. We refined structures of canonical SVV transcripts and uncovered numerous RNA isoforms, splicing events, fusion transcripts and non-coding RNAs, mostly unique to SVV. We verified the expression of canonical and newly identified SVV transcripts *in vivo*, using lung samples from acutely SVV-infected cynomolgus macaques. Expression of selected transcript isoforms, including those located in the unique left-end of the SVV genome, was confirmed by reverse transcription PCR. Finally, we performed detailed characterization of the SVV homologue of the VZV latency-associated transcript (VLT), located antisense to ORF61. Analogous to VZV VLT, SVV VLT is multiply spliced and numerous isoforms are generated using alternative transcription start sites and extensive splicing. Conversely, low level expression of a single spliced SVV VLT isoform defines *in vivo* latency. Notably, the genomic location of VLT core exons is highly conserved between SVV and VZV. This work thus highlights the complexity of lytic SVV gene expression and provides new insights into the molecular biology underlying lytic and latent SVV infection. The identification of the SVV VLT homolog further underlines the value of the SVV non-human primate model to develop new strategies for prevention of herpes zoster.

## Author summary

Varicella-zoster virus (VZV)–a ubiquitous human pathogen–infects most individuals during childhood, leading to chickenpox, after which the virus persists in the host for decades. Later in life, VZV reactivates to cause shingles, frequently associated with difficult-to-treat chronic pain. Our limited understanding of the viral life-cycle hampers the development of more effective treatment options. Simian varicella virus (SVV) is the non-human primate homologue of VZV and causes a natural disease in Old World monkeys with clinical, pathological, and immunological features resembling human VZV infection. However, it is unclear how similar both viruses are at the molecular level. Here, we have revisited the genome-wide transcriptional activity of SVV during lytic infection of kidney epithelial cells derived from two non-human primate species and validated expression of newly identified viral transcripts in lung tissue from SVV-infected animals. Together, this has led to the identification of numerous alternative RNA isoforms, mostly unique to SVV, and some of which may have functional implications for the virus. Notably, we defined the SVV latency-associated transcript, which is highly similar to its VZV counterpart. In conclusion, our study shows the value of understanding the molecular biology of a given animal model and identifies potentially conserved mechanism of latency.

## Introduction

Varicella-zoster virus (VZV) is an endemic human neurotropic alphaherpesvirus and causative agent of both varicella and herpes zoster [1]. Although varicella is typically benign during childhood, primary VZV infection in adults can be accompanied by severe ocular and neurological complications [1]. During primary infection VZV establishes a lifelong latent infection predominantly in sensory neurons of the dorsal root ganglia (DRG) and trigeminal ganglia (TG). In one-third of infected individuals, VZV reactivates from latency to cause herpes zoster, which is often complicated by chronic pain in the affected dermatome [2,3] and can lead to severe disease in immunocompromised patients. As VZV is highly species-specific, no animal models exist that fully recapitulate the viral life cycle of primary infection, latency and reactivation, thus hampering the development of antiviral therapies targeting these viral processes.

The alphaherpesvirus simian varicella virus (SVV) was originally isolated from naturally infected monkeys that presented with a varicella-like disease, and is the closest known relative of VZV. Infection of non-human primates with SVV recapitulates virological, immunological and pathological features of VZV infection in humans [4], providing the opportunity to study the pathogenesis of varicella, latency and herpes zoster *in vivo* [5]. However, the molecular biology of lytic SVV infection is poorly understood. SVV and VZV genomes are co-linear, similar in size and share 68 of the canonical open reading frames (96% of SVV) that are assumed to be functional equivalents [6]. Currently, the SVV transcriptome has been annotated based on uninterrupted coding sequences of at least 75 amino acids that share homology to VZV [6] and only a handful of transcripts are further characterized [7–9]. Recently, we have refined the architecture of most transcriptional units of VZV, and showed that the complexity of the lytic VZV transcriptome was vastly underestimated [10]. Given the close genetic relatedness between VZV and SVV, it is likely that the SVV transcriptome is similarly complex.

During latency, VZV and SVV DNAs persist as a closed circular chromatinized episome in neuronal nuclei, and are generally transcriptionally repressed [11,12]. Recently, we have shown that the latent VZV transcriptome is restricted to the VZV latency-associated transcript (VLT), and, frequently, lower level expression of a fusion product of VLT to the lytic gene ORF63 (VLT-63) [13,14]. During lytic VZV infection, VLT is expressed with late kinetics and a large diversity of transcript isoforms is produced [10]. Similar to latency transcripts of related alphaherpesviruses, VLT is located antisense to the ICP0 homologue, open reading frame 61 (ORF61) [15]. Previous studies demonstrated that SVV expresses a transcript antisense to ORF61 [4,16]. Similar to VZV VLT, this antisense ORF61 transcript is expressed at low levels during lytic infection, but is the most prevalent and abundant transcript expressed during latency in SVV-infected non-human primates. However, the structural characteristics of the SVV antisense ORF61 transcript and its similarity to VZV VLT remain poorly defined.

In this study, we aimed to provide a comprehensive annotation of the lytic SVV transcriptome and to characterize the SVV homologue of VZV VLT during lytic and latent SVV infection.

## Results

### Reannotation of the SVV transcriptome by direct RNA sequencing

Direct RNA sequencing (dRNA-Seq) on nanopore arrays is a powerful technique that can sequence full-length RNAs while simultaneously capturing splice junction usage, transcription starts sites (TSS), and cleavage and polyadenylation site (CPAS) [17,18]. To determine the structure of the SVV transcriptome, we infected African green monkey (AGM) kidney epithelial BS-C-1 cells with wild-type SVV strain Delta for 96 hours, performed dRNA-seq on the polyadenylated RNA fraction and complemented this with short-read Illumina RNA-seq to enable error correction of dRNA-seq reads (S1 Table). To reconstruct the SVV transcriptome we defined transcription units using the identified TSS and CPAS sites and subsequently determined splice sites and alternative transcript isoforms by manual inspection of the read data using IGV [10,17,19]. Consistent with the 3’→5’ direction of dRNA-seq, we identified CPAS for all SVV transcription units. However, some low-abundant transcripts lacked a detectable TSS peak, in which case we inferred the TSS from the 5’ end of the coding sequence (CDS) of the encoded protein product. An example for the annotation strategy is given in S1 Fig, for the RNAs that encode pORF18, pORF19 and pORF20. This area includes a polycistronic transcription unit comprising RNA18 and RNA19, sharing the same CPAS sites, but distinct TSS, and a monocistronic transcription unit of RNA20 defined by a single TSS and a single CPAS. Finally, we analyzed the coding potential of all identified SVV transcripts using CPC2.0 [20], an *in-silico* tool that integrates transcript length, Fickett score and isoelectric point of the protein and ORF integrity to predict its coding probability.

### Reannotation of the SVV transcriptome identifies alternative transcript isoforms and putative noncoding RNAs

**Fig 1:**
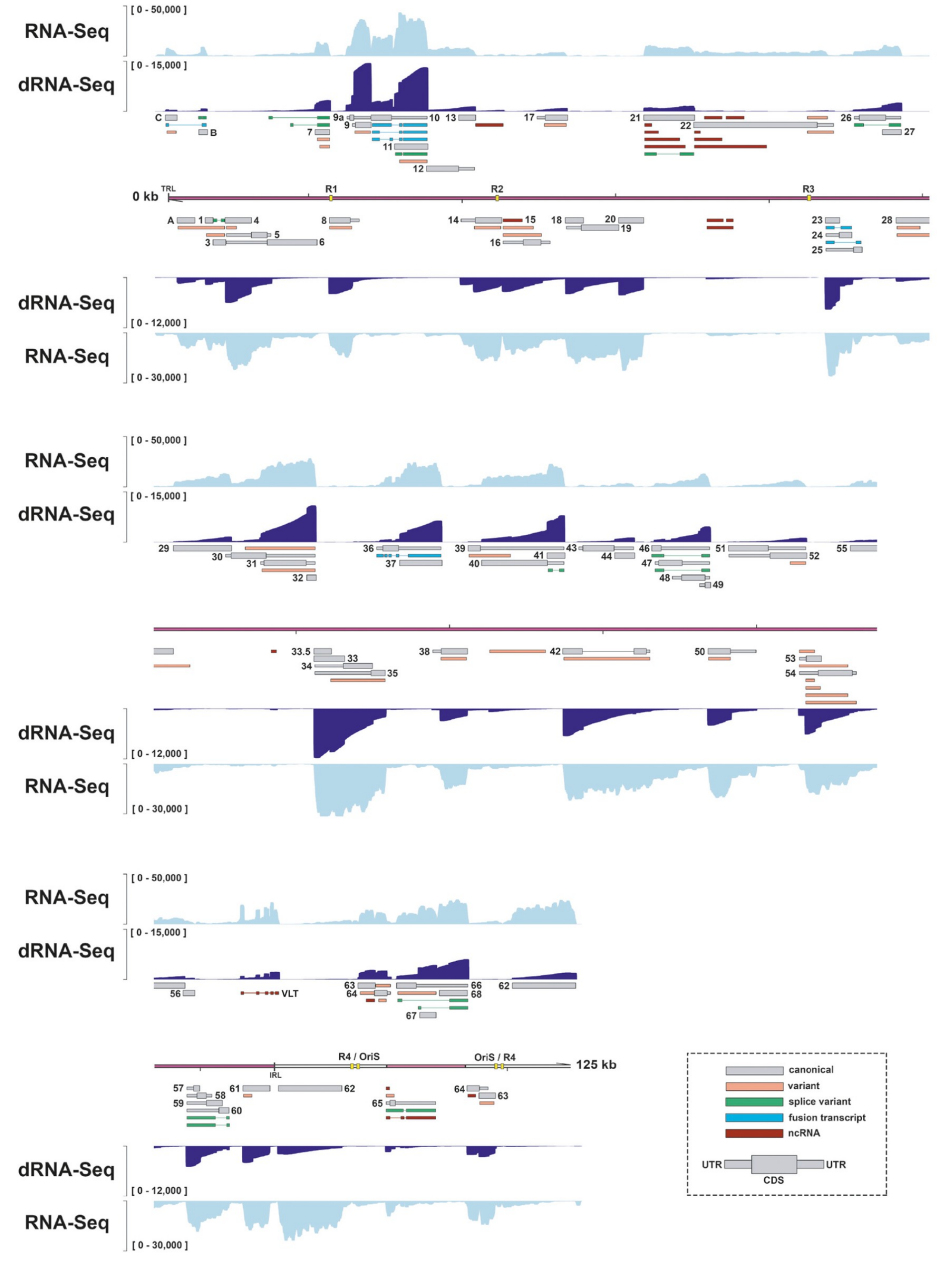
Reannotation of the lytic SVV transcriptome in BS-C-1 cells. The reannotated lytic SVV transcriptome consists of 150 RNAs, 73 of which encode canonical ORFs with UTRs (thin box) and CDS (wide box) indicated in grey, 34 variants RNAs (orange), 15 splice variants (green), 7 fusion transcripts (blue) and 21 putative non-coding RNAs (red). Shown are Illumina RNA-seq (light blue) and nanopore dRNA-seq (dark blue) coverage plots of BS-C-1 cells lytically infected with SVV strain Delta for 96 hours. Maximum read depth per track is given on the Y-axis. Double black lines represent the SVV genome, with the long unique region (UL) in purple fill, short unique region (US) in pink and internal/terminal repeat sequences (IRS/TRS) no fill, ticks indicate 10 kb intervals and the reiterative repeat regions R1 to R4 and both copies of OriS are indicated as yellow boxes on the genome track.

In total, we annotated 150 SVV RNAs that were readily detected in SVV-infected BS-C-1 cells (Fig 1 and S2 and S3 Tables). SVV RNAs were numbered according to the respective canonical ORF and classified into five distinct categories. We identified 73 RNAs that we categorized as ‘canonical’ SVV transcripts: either the only transcript encoding the complete canonical protein or, in case of multiple complete protein coding transcripts, the most abundant by ‘transcript per million’ (TPM). Next, we classified spliced RNAs into two groups: ‘fusion transcripts’ (n = 7) were defined as RNAs that fuse two coding domains of different ORFs through splicing, whereas ‘splice variants’ (n = 15) encompasses all remaining spliced transcripts. Putative ‘non-coding RNAs’ (ncRNAs) (n = 21) were defined as SVV transcripts that had incomplete ORF integrity, or were predicted to be non-coding by CPC2.0. Finally, all remaining truncated or extended isoforms of canonical SVV RNAs were classified as ‘variant RNAs’ (n = 34). A single canonical RNA15-1, encompassing pORF15, was classified as non-coding, because the 3’ end of the transcript does not include the annotated nor another stop codon, resulting in incomplete ORF integrity (S2 Fig). To ascertain that classification of RNA15-1 was not influenced by mutations in our isolate or potential errors in the GenBank sequence in this area, we redefined the SVV DNA sequence by integrating nanopore DNA sequencing and Illumina RNA-seq (S3 Fig and S4 Table). We detected multiple SNPs across the entire genome of our SVV isolate (SVV Delta-EMC) compared to the current GenBank reference sequence (NC_002686.2), but none of these were located in the RNA15 locus (S3 Fig). Notably, comparison of our dRNA-seq derived data with previously published Northern blotting analysis of the SVV RNA14 locus validated the expression of RNA14-1 (1,710 nt) and RNA14-2 (2,580 nt), most likely corresponding to the ±1.9 kb and ±2.5 kb bands on the blots [8].

### Confirmation of selected newly identified SVV transcripts

Reannotation of the SVV transcriptome revealed that lytic SVV gene expression is considerably more complex than previously anticipated, with alternative TSS and splicing events occurring throughout the entire genome. To confirm our dRNA-seq results, we performed RT-PCR and Sanger sequencing analysis on a selection of loci that together encompassed all five categories of SVV transcripts (canonical, variant, splice variant and fusion transcripts, and putative ncRNAs).

**Fig 2:**
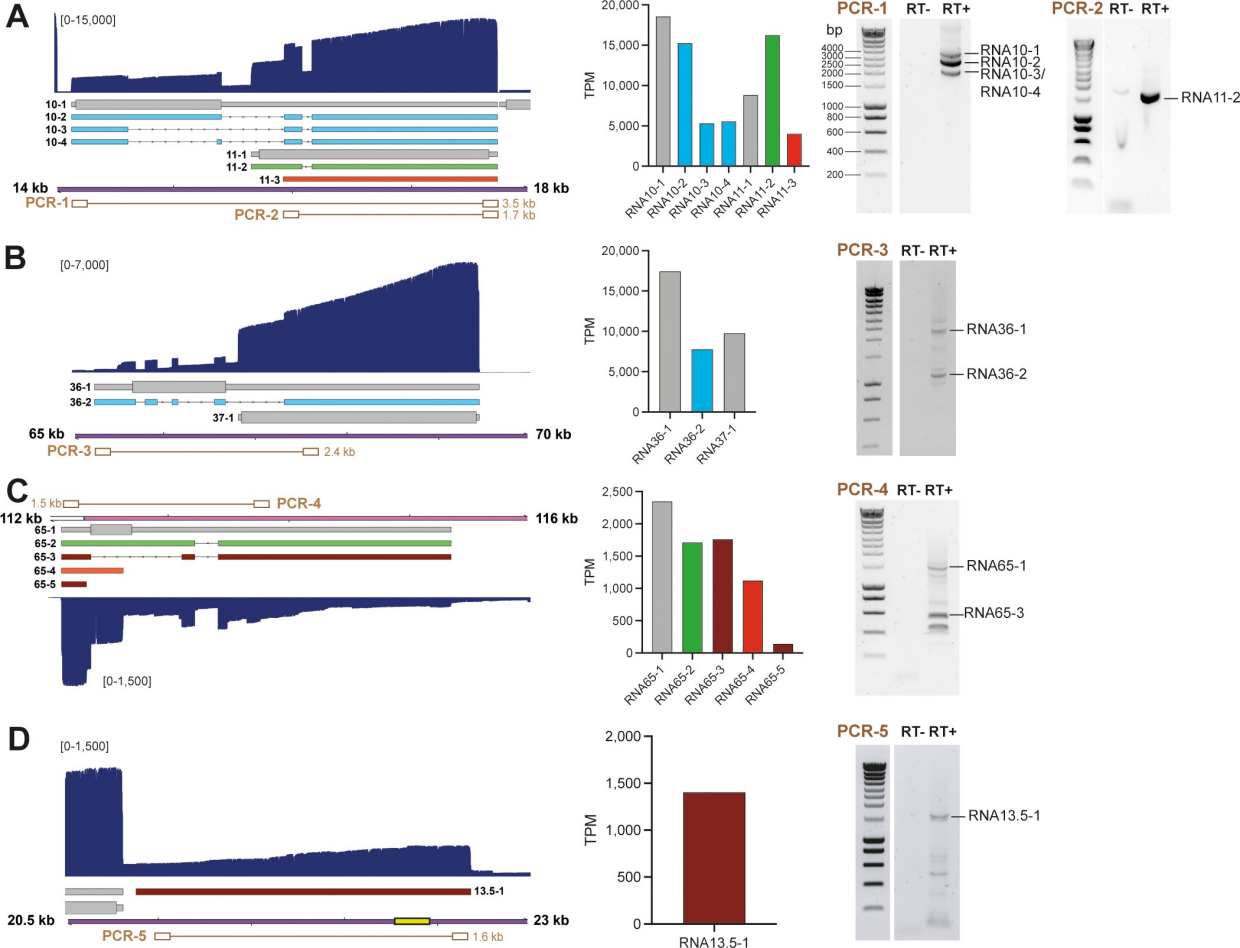
Confirmation of selected novel SVV transcripts by RT-PCR. (A-D) Examples of a polycistronic transcription units encoding canonical transcripts, novel splice variants and fusion transcripts within the RNA10 and RNA11 locus (A) and RNA36 and RNA 37 (B), canonical, spliced and variant isoforms and putative ncRNAs in the RNA65 locus (C) and newly identified putative ncRNA13.5–1 (D). The structure, location and relative expression levels of novel SVV transcripts are shown, together with agarose gel images of RT-PCRs that confirm their expression. Left panels: Nanopore dRNA-seq coverage plots with annotated SVV transcripts of lytically infected BS-C-1 cells. Canonical with CDS (wide box) and UTR (thin box) in (grey), variant (orange), splice variant (green), fusion transcripts (blue) and non-coding (red) RNAs are indicated. Double black lines represent the SVV genome, with UL in purple fill, US in pink and IRS/TRS no fill, ticks indicate 1 kb intervals and the reiterative repeat regions R1 to R4 and both copies of OriS are indicated as yellow boxes on the genome track. Middle panels: bar graphs indicating transcripts per million (TPM) counts for each transcript isoform. Right panels: RT-PCR was performed on RNA extracted from lytically SVV-infected BS-C-1 cells at 96 hpi. RT+/RT-: reverse transcriptase added or omitted during cDNA synthesis. PCR numbers correspond to primer pairs indicated in brown in Figure A-D and in S5 Table. When multiple bands are observed, sequence confirmed bands are indicated.

The polycistronic transcription unit RNA10 and RNA11 encodes for 7 distinct transcripts, including 2 canonical RNAs (RNA10-1 and RNA11-1), 4 splice variant RNAs (RNA10-2, RNA10-3, RNA10-4 and RNA11-2) and one 5’ truncated variant (RNA11-3) (Fig 2A; left panel). Notably, when we determined the relative abundance of each RNA, several alternative isoforms were abundantly expressed, with RNA11-2 to comparable levels as its corresponding canonical transcript RNA11-1. We confirmed the presence of multiple spliced isoforms of RNA10 and the splice junctions shared between RNAs 10–2, 10–3, 10–4 and 11–2 by RT-PCR on RNA isolated from SVV-infected BS-C-1 cells (Fig 2A; right panel).

Similarly, the polycistronic transcription unit comprising RNA36 and RNA37, encoding thymidine kinase and glycoprotein H respectively, also encodes for a novel fusion transcript that fuses part of the coding domains of ORF36 protein (pORF36) into the remainder of pORF37 (Fig 2B). This fusion transcript accounted for approximately 30% of all RNA36 transcripts, and RT-PCR readily detected the unspliced RNA36-1 and spliced RNA36-2.

Finally, pORF65 –a small protein of 77 amino acids–was previously annotated to be encoded by a single RNA [6]. However, our reannotation shows that the CDS of pORF65 is preceded by an unusually long 5’UTR that lies antisense to RNA66 and RNA67 (Fig 1). Furthermore, 4 alternative isoforms are encoded within this locus, two of which are splice variants (RNA65-2 and RNA65-3), and two that are predicted to be non-coding RNAs (RNA65-3 and RNA65-5) (Fig 2C). Again, TPM analysis showed that the canonical RNA65-1, and the two isoforms RNA65-2 and RNA65-3, that use the same TSS, were equally abundant, whereas the other two transcripts, RNA65-4 and RNA65-5, were less abundant. We confirmed the expression of RNA65-1, RNA65-3 and of additional alternatively spliced isoforms of lower abundance (Fig 2C).

The revised SVV transcriptome is predicted to encode seven novel non-coding transcripts–RNA13.5–1, RNA22.5–1, RNA22.5–2, RNA22.5–3, RNA31.5–1, RNA38.5–1 and RNA64.5–1 –that are situated antisense to other transcription units. Whereas most of these putative ncRNAs are unique to SVV and of low abundance, RNA13.5–1 is conserved in VZV and is expressed at relatively higher levels compared to other SVV ncRNAs. We confirmed the expression of RNA13.5–1 using strand-specific RT-PCR and Sanger sequencing (Fig 2D).

### Detailed analysis of the unique leftward terminus of the SVV genome

Although SVV and VZV genomes are colinear and share most of their proteins, a notable exception is a 5 kb region oriented at the extreme 5’ end of the canonical genome arrangement [6]. Whereas this region in VZV encodes for the proteins pORF0, pORF1, pORF2 and pORF3, SVV lacks a homologue of pORF2, but instead encodes for two additional proteins pORFA and pORFC, while VZV pORF0 is homologous to SVV pORFB. SVV pORFA is a truncated version of SVV pORF4 encoded by a 1.0 kb transcript and was shown to be non-essential for virus replication [21]. pORFC is a paralogue of pORFB and is also known as UL56A. The presence of UL56A is rather unique among alphaherpesviruses, with *Psittacid herpesvirus 1* –part of the evolutionary distinct *Iltovirus* genus–being the only other alphaherpesvirus that encodes a UL56A [22]. However, neither the transcript structure nor the protein function of pORFC has been further detailed.

**Fig 3:**
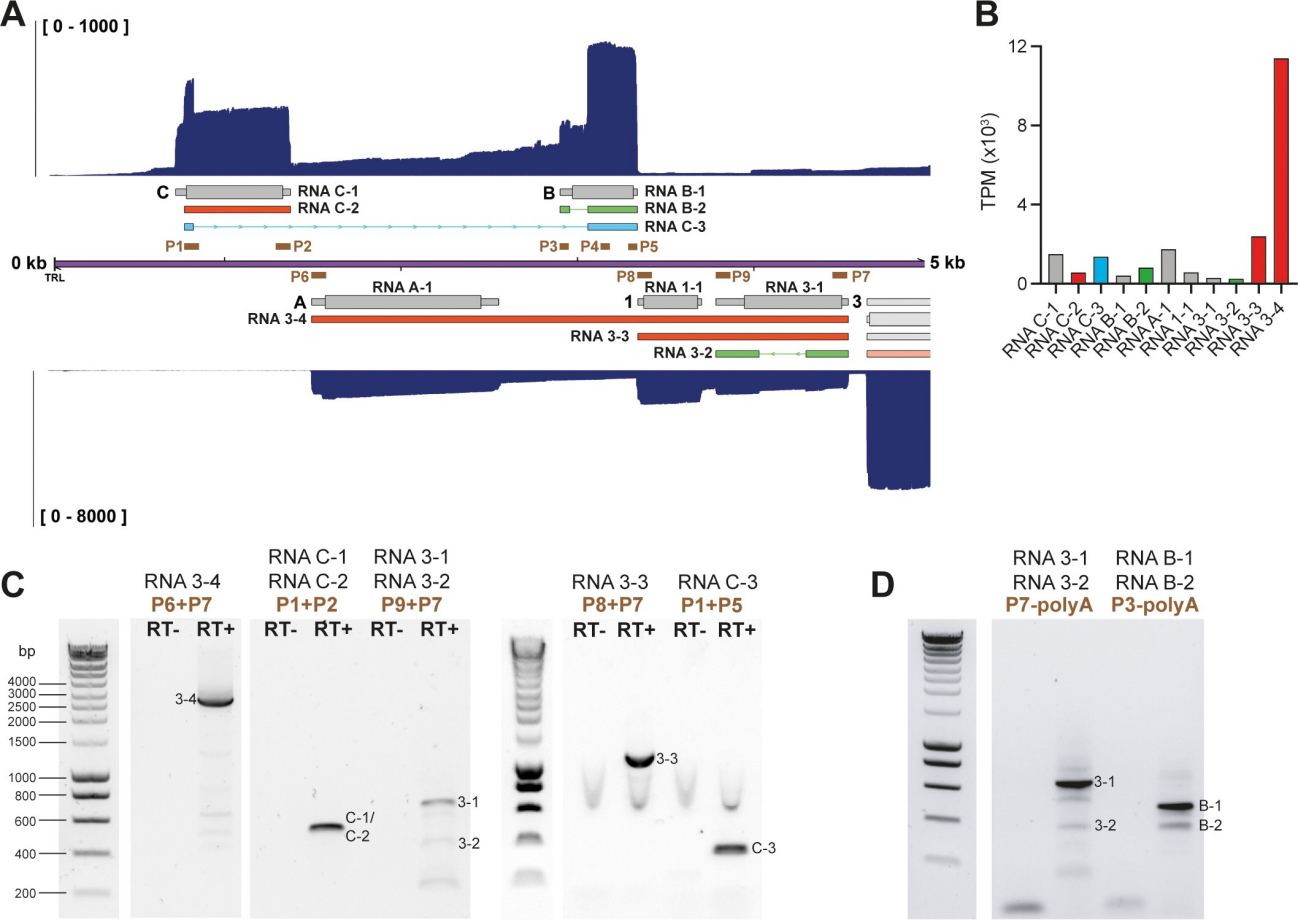
Structure of transcripts expressed from the unique left-end of the SVV genome. (A) Nanopore dRNA-seq coverage plots with annotated SVV transcripts of lytically SVV-infected BS-C-1 cells. Canonical with CDS (wide box) and UTR (thin box) in (grey), variant (orange), splice variant (green), fusion transcripts (blue) and non-coding (red) RNAs are indicated. Double black lines represent the SVV genome, with UL in purple fill, US in pink and IRS/TRS no fill and ticks indicate 1 kb intervals. Primers used for RT-PCR confirmation are indicated as brown boxes. (B) Bar graph indicating transcripts per million (TPM) counts for each transcript isoform. (C-D) RT-PCR on RNA extracted from lytically infected BS-C-1 cells at 96 hpi. When multiple bands are observed, sequence confirmed bands are indicated. RT+/RT-: reverse transcriptase added or omitted in cDNA synthesis. In (D) Strand-specific RT-PCR using a sequence specific forward primer and a poly-A reverse primer. Primer pairs are indicated and sequences are given in S5 Table.

Our reannotation shows that both strands in this region have a more complex organization than previously described. On the forward strand of the SVV genome, five distinct transcripts were identified, with RNAC-1 and RNAB-1 encoding for canonical pORFC and pORFB respectively (Fig 3A). Notably, read-through transcription and subsequent splicing resulted in a relatively abundant fusion transcript RNAC-3 that is predicted to combine the N-terminal 13 aa of pORFC and the C-terminal 85 aa of pORFB (Fig 3B). Next to the canonical transcripts RNA A-1, RNA1-1, and RNA3-1 on the reverse strand of the SVV genome, we identified two variant transcript isoforms that span multiple CDS (RNA3-3 and RNA3-4) and a spliced alternative isoform of RNA3 (RNA3-2; Fig 3A). SVV ORFA, ORF1, ORF3 genes form a polycistronic transcription unit, with most abundant expression of the polycistronic transcripts RNA3-3 and RNA3-4. Alternative TSS and CPAS sites exist that can generate RNAs coding for the individual ORFs, but these are infrequently used (Fig 3B). By RT-PCR and Sanger sequencing, we confirmed the expression of RNAC-1 and/or RNAC-2 and the spliced RNAC-3 (Fig 3C). Strand-specific RT-PCR and sequencing of RNAB-1 and RNAB-2 confirmed the presence of both transcripts (Fig 3D). On the reverse strand, strand-specific RT-PCR and sequencing confirmed the expression RNA3-1 and RNA3-2 (Fig 3D). Finally, we confirmed the expression of RNA3-3, the abundant RNA3-4, several lower expressed variants of the latter, and splicing of RNA3-2 (Fig 3C).

### The lytic SVV transcriptome is similar between infected African green and rhesus macaque derived kidney epithelial cells

**Fig 4:**
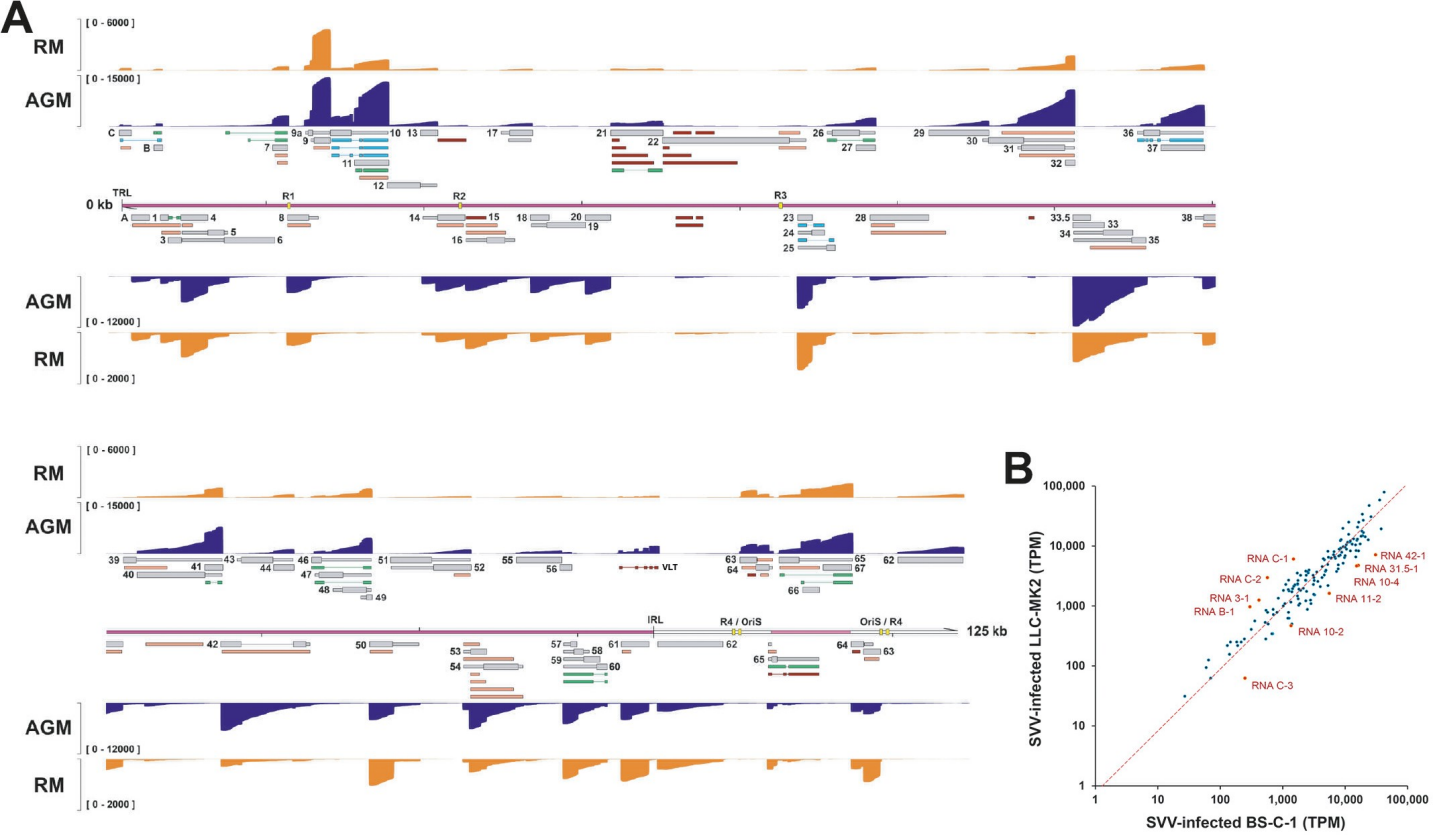
Comparison of the lytic SVV transcriptome in rhesus macaque (RM) derived LLC-MK2 cells and African green monkey (AGM) derived BS-C-1 cells. (A) Coverage plots showing dRNA-seq data of SVV-infected LLC-MK2 (orange) and BS-C-1 (dark blue) cells, with Y-axis indicating read depth per track. Canonical (grey), variant (orange), splice variant (green), fusion transcripts (blue) and non-coding (red) RNAs are indicated. Double black lines represent the SVV genome, with UL in purple fill, US in pink and IRS/TRS no fill, ticks indicate 10 kb intervals and the reiterative repeat regions R1 to R4 and both copies of OriS are indicated as yellow boxes on the genome track. (B) Scatter plot shows correlation between abundance (TPM) of SVV transcripts in LLC-MK2 cells (y-axis) and BS-C-1 cells (x-axis). RNAs differentially expressed (i.e. mean Log2 ratio (“TPM in BSC-1” / “TPM in LLC-MK2”) ± 2 times SD) between BS-C-1 cells and LLC-MK2 cells are highlighted and annotated in red.

Diverse non-human primate species are susceptible to SVV infection, but disease severity varies between species. Notably, SVV infection is more severe in AGM compared to cynomolgus macaques (CM) and rhesus macaques (RM) [4,23,24]. To determine if the SVV transcriptome differs between AGM and RM, we compared the SVV transcriptome at 96hpi in kidney epithelial cells of AGM (BS-C-1) and RM (LLC-MK2) origin (Fig 4). Although the overall experimental read depth was similar between AGM and RM cell types, the SVV read depth was markedly lower in lytically infected LLC-MK2 cells. Nevertheless, we detected most (95%) SVV RNAs that we annotated in lytically infected BS-C-1 cells (Fig 1 and S6 Table). SVV transcripts that were not detected corresponded to those which are low-abundant viral RNAs or isoforms in SVV-infected BS-C-1 cells. Relative SVV transcript counts in BS-C-1 cells significantly correlated with those in LLC-MK2 cells (Fig 4B). Importantly, we did not identify any unique transcripts or transcript isoforms in LLC-MK2 cells, indicating that the lytic SVV transcriptome is consistent across host species. However, the relative expression level of some SVV RNAs differed between BS-C-1 and LLC-MK2 cells (Fig 4B). Interestingly, most differentially expressed SVV transcripts originate from the unique leftward terminus of the SVV genome and the RNA10/11 locus, many of which are (splice) variants of canonical transcripts.

### The SVV transcriptome is similar between lytic SVV infection *in vitro* and *in vivo*

**Fig 5:**
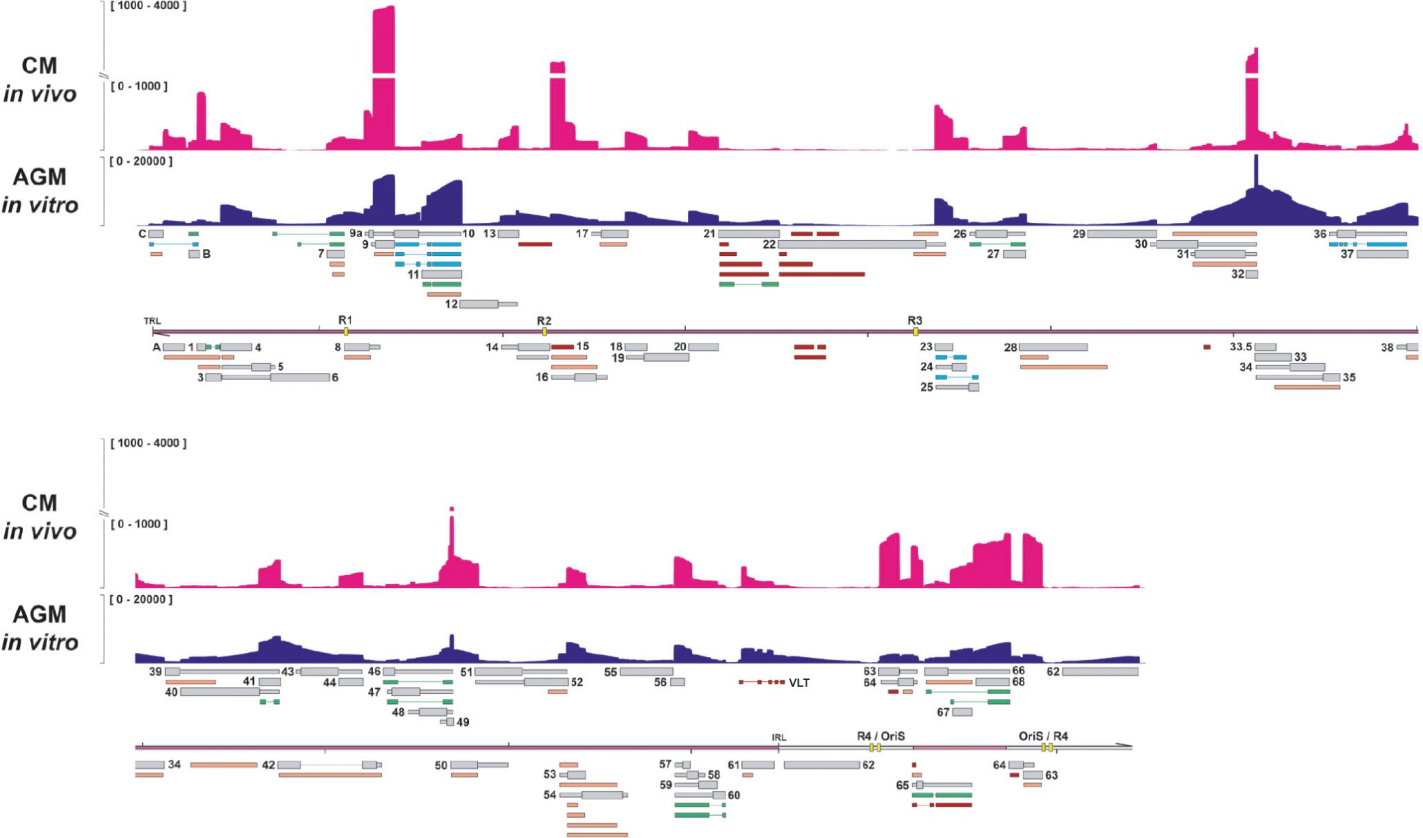
Comparison of the lytic SVV transcriptome *in vitro* and *in vivo*. PCR-cDNA sequencing of RNA obtained from lung tissue of cynomolgous macaques at 3 days post-infection with SVV strain Delta reveals the lytic transcriptome *in vivo*. Coverage plots showing PCR-cDNA-seq of SVV-infected CM lung tissue *in vivo* (magenta) and AGM BS-C-1 cells *in vitro* (dark blue), with Y-axis indicating the unstranded read depth. Canonical (grey), variant (orange), splice variant (green), fusion transcripts (blue) and non-coding (red) RNAs are indicated. Double black lines represent the SVV genome, with UL in purple fill, US in pink and IRS/TRS no fill, ticks indicate 10 kb intervals and the reiterative repeat regions R1 to R4 and both copies of OriS are indicated as yellow boxes on the genome track.

The SVV non-human primate model infection provides the unique opportunity to investigate the viral transcriptome during lytic infection *in vivo*. Thus, we extracted RNA from lungs of intratracheally SVV-infected cynomolgus macaques at 3 days post-infection and determined the structure of the SVV transcriptome. The relatively low yields of viral RNA compared to infected cell cultures necessitated the use of long-read PCR-cDNA sequencing (Fig 5). Unfortunately, this approach obscures stranded information, requiring thorough visual inspection of the read data to determine which RNAs are present, especially when transcripts on both strands overlap. To aid visual comparison, we merged the forward and reverse strand reads of the dRNA-seq data on *in vitro* infected BS-C-1 cells (Fig 5). Similar to the dRNA-seq on LLC-MK2 cells, the majority of the 150 RNAs expressed during lytic SVV infection *in vitro* were readily detectable *in vivo*, whereas low abundant RNAs were absent (S6 Table). Moreover, no unique *in vivo* SVV transcripts or isoforms were observed. However, the relative abundance of certain SVV RNAs differed, with RNAs encoding pORFB, pORF9 and pORF15 overrepresented *in vivo* compared to *in vitro* samples (Fig 5). Interestingly, the VZV and HSV-1 orthologues of pORFB are required for growth *in vivo* [25–28], whereas pORF9 orthologues are abundantly expressed viral proteins that are essential for VZV replication and required for efficient HSV-1 spread [10,29–31]. Collectively, our data demonstrate that the SVV transcriptome is conserved between species and is comparable between lytic SVV infection in *in vitro* and *in vivo*.

### SVV encodes a multiply spliced homologue of VZV VLT

**Fig 6:**
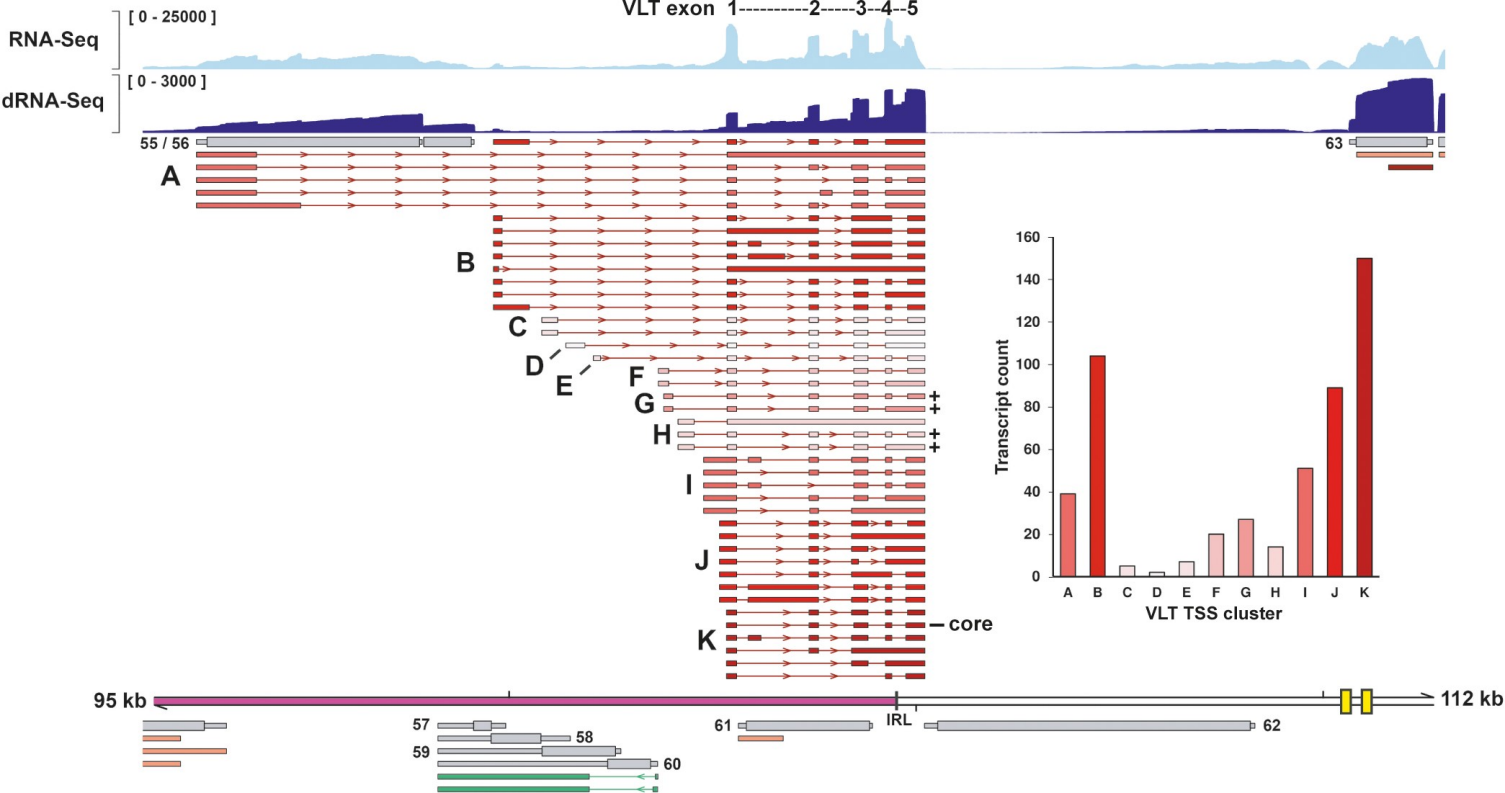
Structure of VLT RNA isoforms expressed during lytic SVV infection of BS-C-1 cells. VLT isoforms are given are clustered by TSS (A-K). Color gradient indicates relative abundance of transcripts originating from each TSS, and transcript counts are given in the bar graph. Counts only include reads starting within 50 bp from an annotated TSS. Coverage plots show Illumina RNA-seq (light blue) and nanopore dRNA-seq (dark blue) data. Maximum read depth per track is given on the Y-axis. Thick double black lines represent the SVV genome, ticks indicate 10 kb intervals and the reiterative repeat regions R1 to R4 and both copies of OriS are indicated as yellow boxes on the genome track. Plus signs indicate isoforms coding for the longest pVLT homologue and the core VLT is indicated.

Decoding the SVV transcriptome through dRNA-Seq allows for detailed analysis of regions of interests; particularly the locus that is transcriptionally active during latency. For VZV, latency is defined by selective expression of VLT and VLT-ORF63 transcripts, that are located partially antisense to ORF61 and are also expressed during lytic infection [10,13,14]. Transcription from the same genomic location has been reported in SVV [16], but the structure of this antisense ORF61 RNA is poorly defined. Unlike the single unspliced transcript mapping antisense to RNA61 previously reported, our dRNA-seq data revealed a plethora of multiply-spliced transcripts in this locus. By analogy to VZV, we designated these transcripts lytic SVV VLT (Fig 6). In contrast to VZV VLT [10,14], we did not observe any read-through transcripts fusing VLT and ORF63 in SVV. Multiple SNPs were detected in the SVV VLT locus of SVV Delta-EMC compared to the GenBank reference sequence (NC_002686.2) by SVV DNA sequencing (S3 Fig and S4 Table), but none of these disrupt potential splice sites or could otherwise explain the absence of VLT-ORF63 RNAs in SVV.

We identified at least 42 distinct SVV VLT isoforms that were readily detected in lytically SVV-infected BS-C-1 cells and clustered these into 11 groups (A-K) based on their TSS usage (Fig 6). Retention of one or more VLT introns was common and essentially all combinations of identified TSS and downstream VLT exons could be detected at low abundance beyond the 42 defined isoforms. Conventional TPM analysis on a vast number of transcript isoforms that share many characteristics is challenging. Instead, we performed a transcript count per TSS cluster and only included transcripts with a 5’ end within 50nt of a defined TSS (Fig 6; inset). Cluster K contained the most reads, and the isoform of highest abundance. Therefore, we defined the exons of the most abundant isoform as the “core” SVV VLT, and numbered VLT exons based on this isoform. Similar to VZV VLT [13], core SVV consists of five exons, of which two exons are located antisense to ORF61.

The core of VZV VLT encodes a small protein of unknown function: pVLT [13,14]. *In silico* translation of all annotated SVV VLT isoforms revealed the presence of a potential SVV pVLT homologue of 145 aa in four VLT isoforms, and of alternative truncated versions of this protein in most of the other isoforms (S4 Fig). Thus, our data demonstrate the presence of an antisense ORF61 transcript during *in vitro* SVV-infection that, like its VZV counterpart, is extensively spliced and encodes multiple isoforms, some of which are predicted to encode for a putative pVLT homologue.

### SVV VLT is expressed during lytic infection *in vitro* and *in vivo*

**Fig 7:**
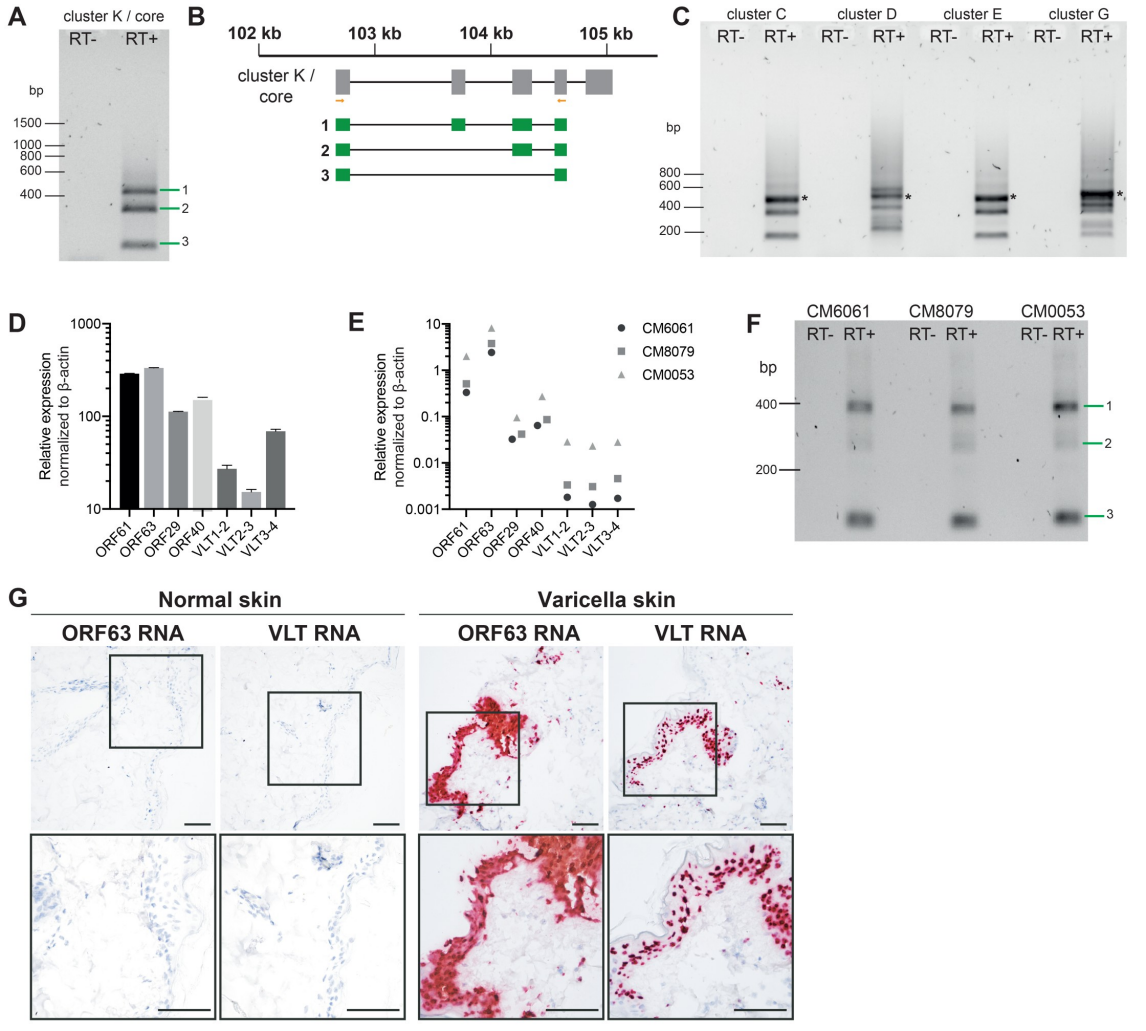
Confirmation of SVV VLT expression during lytic infection *in vitro* and *in vivo*. (A) RT-PCR confirmation of the core of SVV VLT indicates 3 major isoforms in BS-C-1 cells. (B) Diagram showing sequence confirmed SVV VLT isoforms. Grey boxes indicate core VLT according to dRNA seq, orange bars primer locations, and green boxes sequences obtained from indicated bands. (C) RT-PCR confirmation of 4 upstream exons represented by cluster C, D, E and G (see Fig 6) in BS-C-1 cells. Most abundant bands (*) were confirmed by sequencing. (D-E) RT-qPCR was performed on RNA extracted from SVV-infected BS-C-1 cells at 96 hpi (n = 2, mean ± SEM) (D) and lung tissue from n = 3 CM at 3 days post-infection (E). (F) RT-PCR and sequencing confirm presence of multiple isoforms during *in vivo* lytic infection, major bands confirm core exons and isoforms corresponding to B. (G) Detection of VLT and ORF63 RNA (red signal) by *in situ* hybridization in consecutive sections of normal and varicella skin lesions of an SVV-infected AGM at 9 dpi. Sections were counterstained with hematoxylin. Scale bars indicates 50 μm. Representative images are shown for AGM 269.

Given that most SVV VLT isoforms contain the core exons, we first confirmed the expression of spliced VLT during lytic infection of AGM BS-C-1 (Fig 7A and 7B) cells and RM LLC-MK2 cells (S5A Fig) by RT-PCR and Sanger sequencing using primers located in VLT exon 1 and exon 4. Next, we performed RT-PCR from 4 alternative upstream TSS, representing clusters C, D, E and G (Fig 6), and confirmed the presence of multiple alternative VLT isoforms that use these TSS. For each, we sequenced the most abundant band and identified that this was the fusion of an upstream exon to the SVV VLT core in BS-C-1 cells (Fig 7C) and LLC-MK2 cells (S5B Fig). Finally, we determined the relative abundance of SVV VLT in infected BS-C-1 (Fig 7D) and LLC-MK2 (S5C Fig) cells by qPCR with 3 primer-probe sets that span the exon junctions of core VLT, along with the relative expression of 4 other SVV genes. Similar to its VZV counterpart [13], SVV VLT is of relatively low abundance, although the expression levels differed between exon-junctions examined.

RNA-seq analysis of lung tissue from SVV-infected cynomolgus macaques (Fig 5), yielded only a few spliced reads within the SVV VLT locus, hindering detailed analysis of the VLT structure. Therefore, we performed qPCR for SVV VLT and other viral targets on RNA isolated from lung tissue obtained from 3 SVV-infected CMs at 3 dpi [24]. VLT was expressed in all animals at relatively low levels compared to other lytic genes (Fig 7E). Next, we performed RT-PCR for VLT core exons to assess transcript diversity *in vivo*. In contrast to lytic SVV infection *in vitro*, we did not detect many additional splice variants *in vivo*, and Sanger sequencing of the major PCR product confirmed the presence of the VLT core (Fig 7F). Finally, RNA *in situ* hybridization (ISH) was used to confirm the expression of SVV core VLT RNA in lytically infected keratinocytes, which also expressed ORF63 RNA, in skin lesions of SVV-infected AGM (Figs 7G and S5D). In summary, these data confirm the expression of core SVV VLT RNA during lytic infection *in vitro* and *in vivo*.

### VLT is expressed in ganglia during acute and latent SVV infection

**Fig 8:**
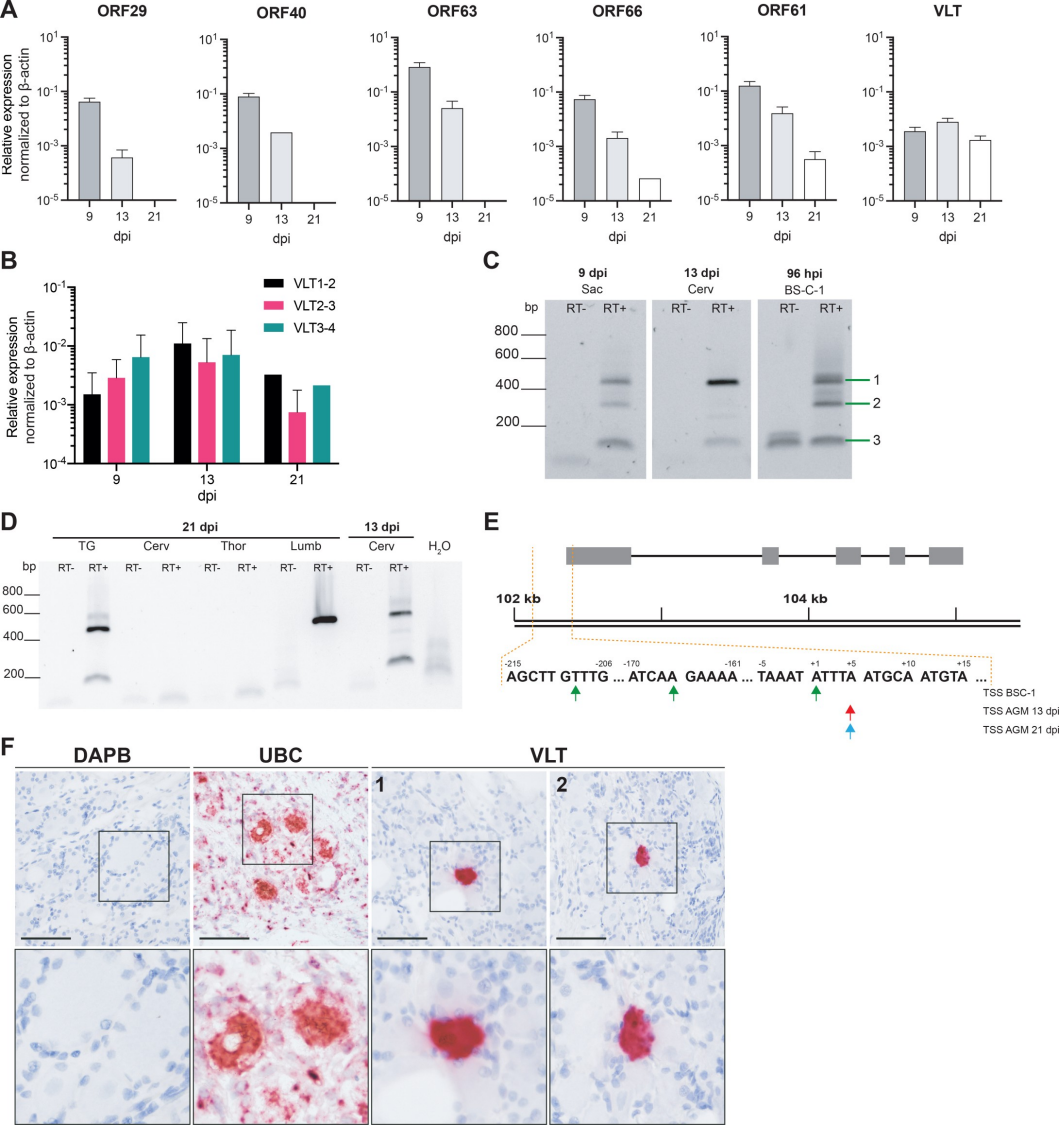
Characterization of VLT expression in ganglia during establishment of latency. (A-B) Detection of lytic SVV RNAs and VLT by qPCR on cDNA obtained from pooled DRG (n = 5 anatomical levels) of SVV-infected AGMs at 9 (n = 2 animals), 13 (n = 2 animals) and 21 (n = 1 animal) days post-infection. Data represent mean ± SEM. (B) Comparison of three VLT exon-spanning primer-probe pairs. (C) RT-PCR showing VLT expression during lytic infection in cell culture (BS-C-1) at 96 hpi, acute sacral (Sac) DRG infection (9 dpi) and transition to latency in cervical ganglia (13 dpi) using primers located in SVV VLT exon 1 and exon 4. Band numbers correspond to isoforms depicted in Fig 7B. (D) Nested RT-PCR on trigeminal ganglia (TG) and DRG (Cerv = cervical, Thor = thoracic, Lumb = lumbar) obtained from SVV-infected AGM at 13 and 21 dpi, respectively. (E) Transcription start sites of VLT in BS-C-1 cells (top row) or AGM ganglia at 13 dpi (middle row) and 21 dpi (bottom row), as determined by 5’RACE. (F) Detection of VLT RNA (red signal) by *in situ* hybridization in DRG neurons of a SVV-infected rhesus macaques at 21 dpi. Probes directed to bacterial gene DAPB and abundantly expressed ubiquitin C (UBC) were included as negative and positive controls, respectively, and sections were counterstained with hematoxylin. Representative images are shown for RM 2207 (lumbar; DAPB, UBC and VLT image 1) and RM 9021 sacral (VLT image 2). Scale bar indicates 50 μm, magnification 40x (upper), inset: 2.5x digital zoom (lower).

During primary lytic infection, VZV and SVV gain access to the sensory neurons of ganglia located along the entire nerve-axis, silence lytic gene expression and ultimately establish neuronal latency. Whereas human TG only offer a static picture once VZV latency has long been established, the SVV model provides the unique opportunity to monitor viral gene expression in ganglia at different stages of infection and latency. Therefore, we performed qPCR on RNA isolated from ganglia of SVV-infected AGM euthanized at 9 dpi, 13 dpi (both: n = 2 animals/time point) and 21 dpi (n = 1 animal) [23]. For each animal ganglia from the left side of the body were pooled based on their anatomical location (cervical, thoracic, lumbar and sacral DRG), generating 4 pooled DRG RNA samples and 1 TG RNA sample per animal. We observed lytic SVV gene expression in ganglia during acute infection, which was decreased at 13 dpi compared to 9 dpi, whereas VLT expression remained stable between 9 and 13 dpi (Fig 8A). Further delineation of VLT expression showed that all three splice junctions were consistently detected, and that VLT was also expressed in AGM ganglia at 21 dpi (Fig 8B). RT-PCR and sequencing confirmed the presence of multiple isoforms at 9 dpi, similar to BS-C-1 cells (Fig 8C), and a single dominant isoform encoding the spliced core VLT at 13 dpi (Fig 8C). To enable detection of VLT at low levels in ganglia of limited availability and small sample size, we designed a sensitive nested PCR approach and showed that in latently SVV-infected ganglia at 21 dpi VLT was predominantly expressed as a single spliced core transcript (Fig 8D).

To further refine the structure of latent SVV VLT, we performed 5’ rapid amplification of cDNA ends (RACE) on RNA obtained from pooled AGM ganglia at 13 dpi and 21 dpi, and RNA from lytically SVV-infected BS-C-1 cells at 96 hpi. Consistent with dRNA-seq results, 5’ RACE indicated multiple TSS for lytically infected BS-C-1 cells in this area: one belonging to cluster H and two to cluster I (Fig 8E). By contrast, a single distinct TSS was detected in ganglia at both 13 and 21 dpi that corresponded to the TSS of cluster I (Fig 8E). Finally, we confirmed by RNA ISH that VLT RNA is expressed in SVV-infected neurons, but not non-neuronal cells, in DRG obtained from SVV-infected rhesus macaques at 21 dpi (Fig 8F).

## Discussion

During the last decade, advances in RNA/cDNA sequencing methodologies have revealed that viral transcriptomes are more complex than previously anticipated, altering our understanding of the molecular biology during all stages of viral infection. Although at the level of the whole organism the SVV non-human primate model mimics VZV disease in humans, it is uncertain to what extent this similarity extends to the molecular level. Therefore, we performed dRNA-seq to examine the architecture of the SVV transcriptome during lytic infection. We present a comprehensive analysis of TSS and CPAS for all protein coding SVV transcripts, defined their 5’ and 3’ UTRs and their diverse isoforms. We show that SVV, like the related alpha-herpesviruses VZV and HSV-1 [10,17] employs read-through transcription, alternative TSS usage, and splicing to diversify its transcriptome and predicted proteome. Finally, we identified the SVV homologue of VZV VLT and show that it is multiply-spliced and expressed during lytic infection and *in vivo* latency.

*Alphaherpesvirinae* share most of their genes, and only a few novel genes have emerged since their last common ancestor. Similar to other double-stranded DNA viruses, most recently evolved genes are located around the genome termini [32–34], in herpesviruses particularly the left-end of the genome [35]. Conversely, although SVV and VZV genomes are largely colinear, their 5’ ends are notably different [6]. Diversification of the gene content of eukaryotes and their associated viruses is likely to occur via gene duplications and subsequent divergence [35,36], evidenced here by the lack a homologue of VZV ORF2 in SVV, and the presence of an additional paralogue of SVV ORFB. Notably, we observed clustering of SNPs in the left-end of the SVV genome suggesting ongoing variation and evolution in this locus (S3 Fig). Previous studies have detailed gene expression and protein production from this SVV locus [21,37], but failed to recognize most of the RNA isoforms. We have shown that gene expression in this locus is complex, variable between RM and AGM cell lines, and characterized by long polycistronic RNAs and splicing. As this is the major site of evolution in herpesviruses, it would be interesting to determine whether these RNA isoforms are essential to the virus and if these ultimately contribute to functional diversification of proteins.

**Fig 9:**
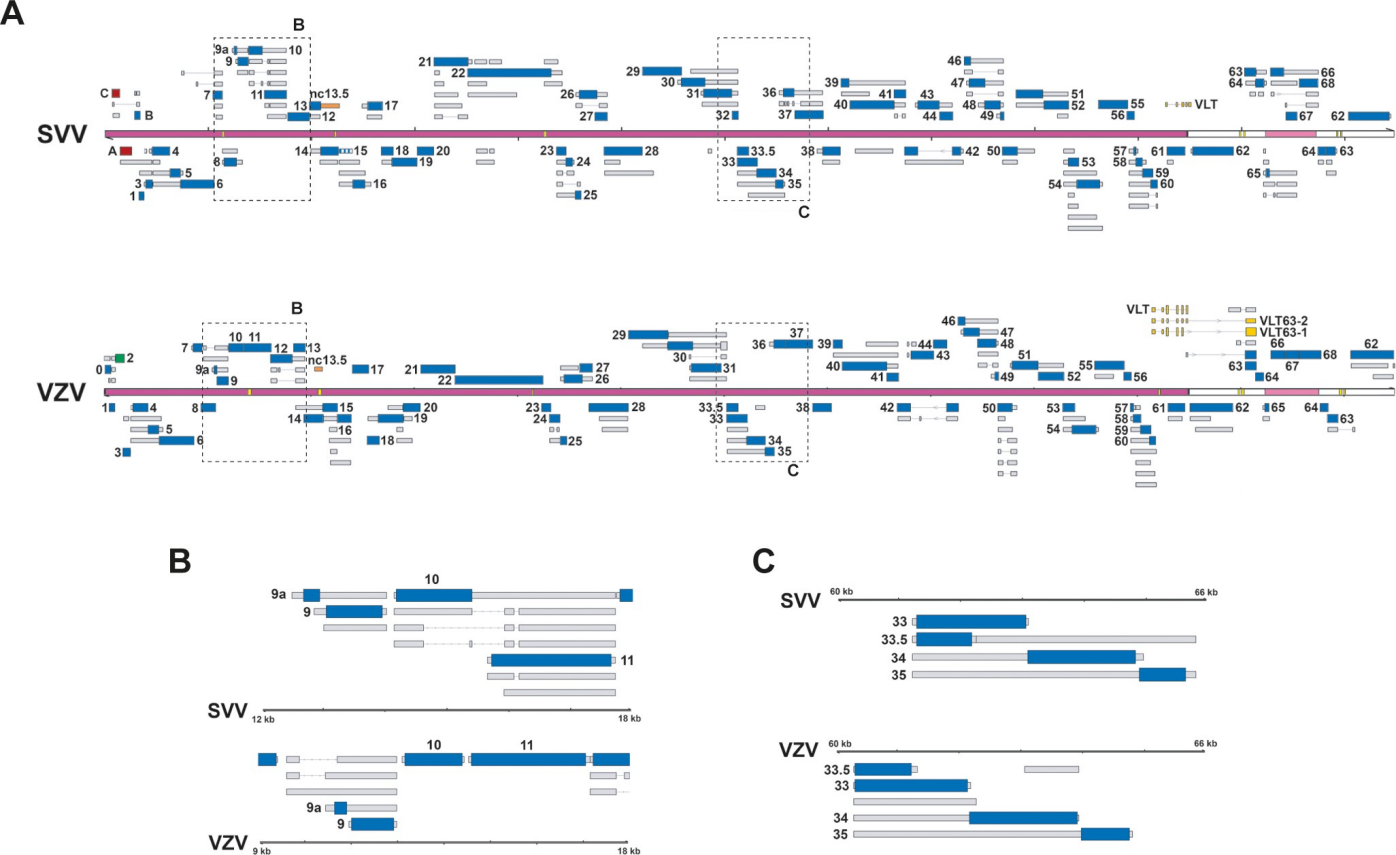
Genome-wide comparison of transcript structures in SVV and VZV. (A) Alignment of SVV and VZV genomes with their respective annotation of the transcriptome. Canonical ORFs in both viruses are highlighted in blue, with UTRs (thin box, grey) and CDS (wide box), whereas transcript isoforms are given in grey. Unique SVV ORFs are indicated in red, unique VZV ORFs in green, conserved ncRNA 13.5 in orange and VLT in yellow. Double black lines represent the SVV/VZV genome, with the long unique region (UL) in purple fill, short unique region (US) in pink and internal/terminal repeat sequences (IRS/TRS) no fill, and the reiterative repeat regions R1 to R4 and both copies of OriS are indicated as yellow boxes on the genome track. Dashed boxes indicate areas shown as enlargement in (B) and (C). (B) Selected region of RNA9-11 in both viruses where transcript structures differ. (C) Selected region of RNA33-35 in both viruses where transcript structures are similar.

Decoding the architecture of the lytic SVV transcriptome enables the comparative analysis between newly annotated SVV and VZV transcriptomes (Fig 9A). With exception of the extreme left-end of the genomes, SVV and VZV genomes are co-linear and share at least 69 protein-coding sequences, as well as one putative ncRNA (ncRNA13.5). However, conserved CDS are often flanked by virus-specific 5’- and 3’-UTR sequences, resulting in modest conservation in transcript structures between both viruses. Moreover, the complexity of specific loci (e.g. the spectrum of alternative isoforms) is often discordant between SVV and VZV. For instance, the RNA9-11 region (Fig 9B) contains a multitude of RNA9A and RNA9 transcripts variants that are specific to VZV (and not SVV) while for SVV there are multiple transcript isoforms of RNA10 and RNA11 that are not observed for VZV. This contrasts with genomic regions that show very high levels of similarity, highlighted here by the RNA33-35 locus (Fig 9C). The importance of these species-specific differences in the biology of these viruses and their respective hosts is not yet known but remains an active area of study.

SVV infects a broad range of Old World monkey species, including Patas monkeys, African green monkeys, and rhesus or cynomologous macaques, and causes disease with clinical, pathological and immunological features that mimics human VZV infection [23,38–40]. Although disease severity varies between species, we showed by dRNA-seq of kidney epithelial cells derived from different non-human primate species that the SVV transcriptome is conserved between species and no species-specific SVV transcripts were detectable. This is similar to our observations for VZV, where we detected no virus strain-specific transcripts, nor any differences between infected neurons or epithelial cells [10]. It is also worth noting that in common with VZV [10] and HSV-1 [17], our approach to defining transcript boundaries can be considered conservative with many additional putative TSS rejected due to their low abundance. We would thus anticipate that continuing studies of all three viruses may yield additional defined transcripts in the future and existing transcriptome annotations may still expand further. There also remains a question as to whether mature non-adenylated viral RNAs such as lariat introns (e.g. HSV-1 LAT, [41]) and circular RNAs (e.g. KSHV circvIRF4, [42]) are also generated for VZV and SVV.

Importantly, the SVV non-human primate models provides a unique opportunity to compare the SVV-transcriptome between *in vitro* and *in vivo* SVV-infections, and thus to validate the use of immortalized cells to annotate viral transcriptomes. Notably, the architecture of the lytic SVV transcriptome was comparable between lung samples from acutely SVV-infected CM and AGM kidney epithelial cells in vitro. However, we observed marked differences in expression levels when compared to *in vitro* lytic infection, especially for RNAs 0, 9, 15, 16, 32, 63 and 65. This is partially in concordance with an earlier study that identified RNAs 9, 15, 16, 57 and 62 as the most abundant SVV transcripts detected in infected T cells in BAL fluid from rhesus macaques [43]. The host factors that potentially regulate these differences in gene expression between *in vitro* and *in vivo* lytic infection remain to be determined. Collectively, this data suggests that cell-type or species-specific host factors regulate differential expression levels of certain SVV transcripts and perhaps even clinical disease severity.

VZV latency is defined by the expression of the multiply spliced VLT and the VLT-ORF63 fusion transcripts, that are located partially antisense to the immediate-early gene ORF61 [13,14]. Similarly, an earlier study identified expression of an unspliced transcript located antisense to ORF61 during lytic SVV infection and particularly during latency [16]. In this study, we have unraveled the structure of this antisense ORF61 transcript and demonstrate that it is a genuine homologue of VZV VLT, and we therefore designated it SVV VLT. Similar to VZV VLT, a plethora of SVV VLT isoforms are generated by extensive splicing, exon skipping and the use of alternative TSS during lytic infection. Interestingly, we observed intron retention in many SVV VLT isoforms, whereas this is absent in VZV VLT. Intron retention is a form of alternative splicing that occurs in transcriptomes of many mammals and contributes to the control of transcript levels through non-sense mediated decay [44]. Alternatively, as a previous study has shown that 6–8% of primate transcripts display differential splicing patterns compared to their human counterparts [45], the mere species difference may dictate splicing efficiency of the VLT locus. Notably, some lytic SVV VLT isoforms encode for a putative protein that shares homology to VZV pVLT, suggesting that despite differential splicing patterns, protein-coding capacity could be conserved.

During primary infection, VZV and SVV gain access to sensory neurons in the TG and DRG to establish lifelong latency. However, it remains unclear when latency is established, and whether it is preceded by lytic gene expression in neurons. Previously, we have shown that SVV DNA and RNA were absent in peripheral tissues of rhesus macaques at 21 dpi, except for restricted low-level gene expression in ganglia, consistent with the establishment of latency [40]. Indeed, we show here that lytic SVV gene expression in AGM is progressively silenced, with an ~100-fold reduction in expression from day 9 post-infection towards 13 dpi. Conversely, expression of SVV VLT remained constant, suggesting that its expression is not actively silenced and hints towards a role for VLT in SVV latency establishment. By 5’ RACE and RT-PCR on ganglia at 13 and 21 dpi, we determined the structure of latent VLT and detected a single isoform, that contains the core of lytic SVV VLT and is consistently spliced. Our findings are consistent with previous studies reporting that SVV gene expression is highly limited and mostly restricted to transcripts originating from the ORF61/VLT locus in latently infected RM and Vervet monkeys [16,46]. Collectively, these data suggest that–like VZV–SVV latency is associated with the expression of a single isoform of VLT.

**Fig 10:**
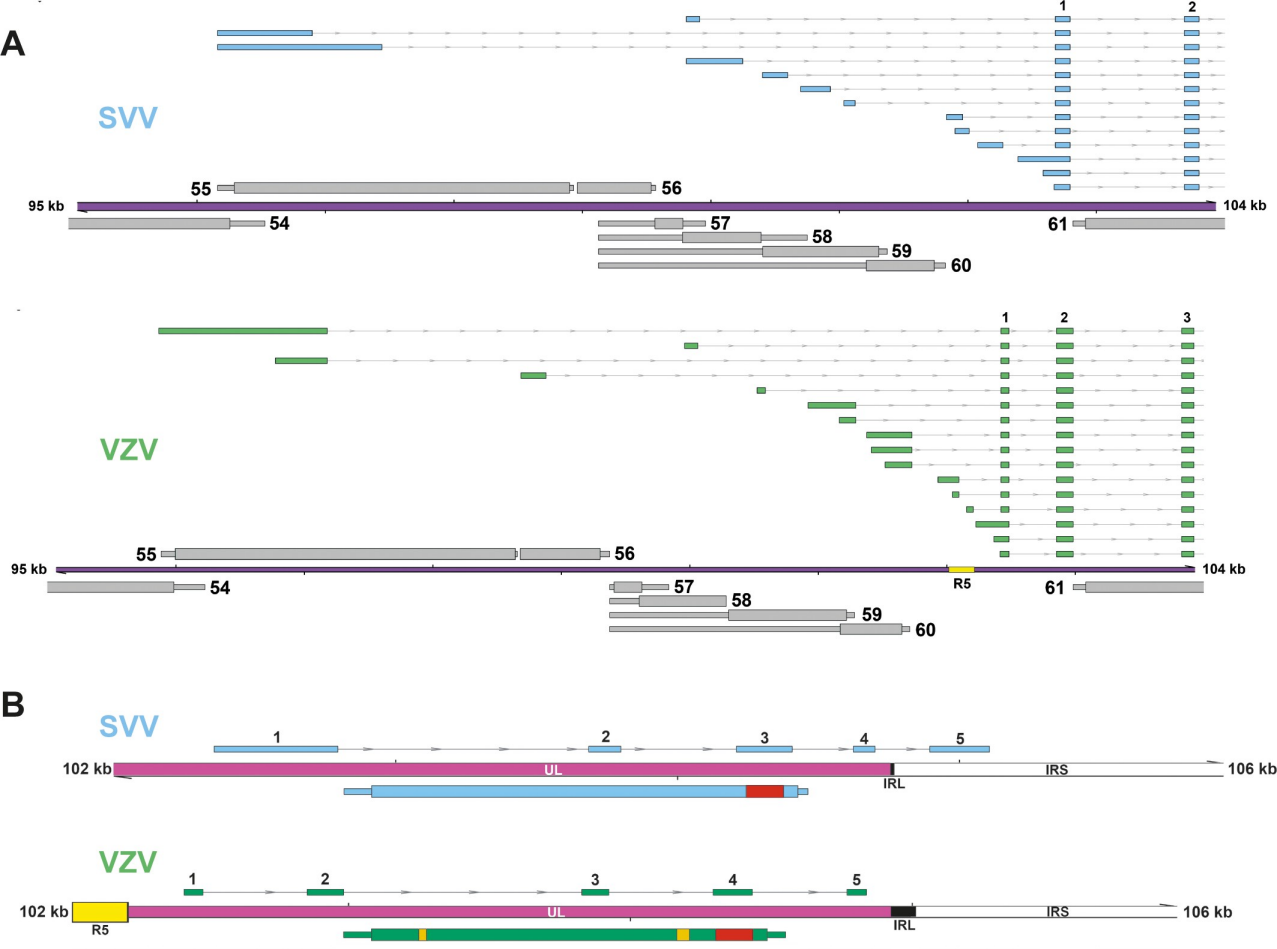
VLT is highly conserved between SVV and VZV. (A) Comparison of the genomic location of SVV (blue) and VZV (green) most abundantly used upstream VLT exons. (B) Comparison of SVV (blue) and VZV (green) core VLT location and structure with respect to other genomic features, such as the IRL/IRS, and ORF61 on the opposite strand. Red box and yellow boxes indicate sequences encoding the RING and SIM domains of VZV pORF61.

VLT is highly conserved between SVV and VZV, enabling an in-depth comparison of this locus. For both viruses, lytic VLT transcription is extremely complex compared to the rest of their transcriptome, with the use of numerous alternative TSS to diversify VLT isoform expression. Comparison of the genomic location of the most abundantly used upstream exons revealed substantial overlap between both viruses (Fig 10A), although the 5’ region of VZV VLT is slightly larger due to the presence of the reiterative repeat region 5 (R5). Subsequent analysis of conserved TSS in genomes of both viruses may provide valuable insight into cell-type specific regulatory elements. Future studies are required to identify and functionally characterize these regulatory sequences. Likewise, VZV VLT and SVV VLT cores exhibit remarkable similarities in structure and location, but their nucleotide or amino acid sequence homology is similar compared to flanking genes. Specifically, the genomic location of 4 VLT exons is conserved with respect to ORF61: one exon situated antisense the 3’UTR of ORF61, 2 exons antisense the CDS of ORF61, of which one lies antisense the encoded RING domain, and 1 exon upstream of ORF61 (Fig 10B). Some differences are also notable; the presence of R5 might have allowed for the formation of the additional core exon in VZV VLT into which all upstream transcription start sites splice, but this exon is not essential to the virus [13]. Whereas VZV VLT either terminates before the IRL, SVV VLT crosses the IRL and has one exon located in the IRS. Furthermore, read-through transcription from VLT into ORF63 occurs in the majority of VZV VLT transcripts, but is absent in SVV VLT. VZV VLT63 encodes for a pVLT-ORF63 fusion protein that can reinitiate viral gene expression from latent VZV genomes *in vitro* [14]. Whether SVV developed alternative strategies to initiate reactivation or selectively expresses a homologue of VLT63 during reactivation or in sensory neurons remains to be determined.

Together, this study enhances the understanding of SVV infection at the molecular biology level, and accentuates the complexity of lytic SVV gene expression. The identification of SVV VLT creates an opportunity to better understand its role in the SVV non-human primate model *in vivo* and provides new opportunities for the development of novel intervention strategies for the treatment or prevention of herpes zoster induced pathologies.

## Materials and methods

### Ethics statement

All nonhuman primate samples were derived from previously published studies and no new experiments were performed [23,24,40].

### Cells and viruses

African green monkey (AGM) kidney epithelial BS-C-1 cells [American Tissue Type Culture (ATCC) no. CCL-26] and rhesus macaque kidney epithelial cells LLC-MK2 cells [ATCC no. CCL-7] were cultured in DMEM (Lonza) containing 10% heat-inactivated fetal bovine serum (FBS; Sigma) and penicillin-streptomycin/L-Glutamine (Lonza). Low-passage wild-type SVV Delta herpesvirus strain was isolated from an acutely infected AGM [23] and propagated < 15 passages in BS-C-1 cells (SVV Delta-EMC).

### Nonhuman primate specimens

Formalin-fixed and paraffin-embedded (FFPE) skin and ganglia samples were obtained from acutely SVV-infected AGM at 9 dpi and latently infected Chinese rhesus macaques at 21 dpi [23,40]. RNA samples were obtained from pooled DRG and TG of SVV-infected AGM at 9, 13 and 21 dpi [23], as well as lung tissue from SVV-infected cynomolgus macaques at 3 dpi [24].

### Preparation of Nanopore direct RNA sequencing libraries and analyses

Nanopore dRNA-Seq libraries were prepared for each sample using between 500–1,000 ng of poly(A) RNA that was previously isolated from 30–50 μg of total RNA using the Dynabeads mRNA Purification Kit (Invitrogen, 61006). The poly(A) RNA was spiked with 0.5 μL of a synthetic Enolase 2 (ENO2) calibration RNA (Oxford Nanopore Technologies Ltd.) and dRNA-Seq libraries prepared according to the standard SQK-RNA002 protocol (Oxford Nanopore Technologies Ltd.). A Nanopore SQK-PCS109 PCR-cDNA library was prepared, according to manufacturer’s instructions, using 200ng of total RNA collected from an *in vivo* SVV-infected CM. Sequencing was performed on a MinION MkIb using R9.4.1 (rev D) flow cells (Oxford Nanopore Technologies Ltd.) for 18–22 hrs (one library per flowcell). Raw fast5 datasets were then basecalled using Guppy v3.6.0 (-f FLO-MIN106 -k SQK-RNA002 / SQK-PCS109) with only reads passing filter used for subsequent analyses. Sequence reads were aligned against the SVV reference genome (NC_002686.2), using MiniMap2 [47] (-ax splice -k14 -uf—secondary = no), with subsequent parsing through SAMtools and BEDtools [48,49]. Here sequence reads were filtered to retain only primary alignments (Alignment flag 0 (top strand) or 16 (bottom strand)).

Transcription start sites (TSS) as well as cleavage and polyadenylation sites (CPAS) were identified using a peak-calling strategy [10,50]. Briefly, BAM files containing aligned reads were parsed using BEDTools to produce strand-separated BED12 files after which each read was truncated to its most 5′ or 3′ alignment position for TSS and CPAS identification, respectively. Peak regions were identified using the HOMER findpeaks module (-o auto -style tss) using a–localSize of 100 and 500 and–size of 15 and 50 for TSS and CPAS, respectively. TSS peaks were compared against Illumina annotated splice sites to identify and remove peak artifacts derived from local alignment errors around splice junctions. Further, we implemented a conservative approach to defining transcript boundaries and thus rejected any TSS within a transcription unit if its depth of coverage was less than 10% of the depth of coverage of the largest TSS in the same transcription unit.

RNA abundance counts were estimated by realigning sequence reads against our updated SVV transcriptome annotation using parameters optimized for transcriptome alignments (minimap2 -ax map-ont -p 0.99). RNA abundance counts were generated by counting alignments against a given RNA only if the alignment 5′ end was located within the first 50 nt of the RNA and the alignment was not marked as supplementary. Transcript per million (TPM) counts were generated by dividing the RNA abundance count for a given transcript by the total number of sequence reads (one read = one RNA) present in the data set and subsequently multiplying by 1 million.

CPC 2.0 ([20]) was used to examine the coding potential of all SVV RNAs defined in this study (S3 Table). Note that RNAs were excluded from CPC 2.0 analysis and defined as putatively noncoding if no proteins greater than 50 amino acids in length were encoded.

### Preparation of stranded Illumina RNA-Seq libraries

Stranded RNA libraries were prepared from poly(A)-selected RNA using the NEBNext Ultra II Directional RNA Library Prep Kit for Illumina (New England Biolabs) and sequenced using a NextSeq 550. Sequence reads were trimmed using TrimGalore (https://www.bioinformatics.babraham.ac.uk/projects/trim_galore/) (—paired—length 30 –quality 30) and aligned against the SVV reference genome using BBmap (https://sourceforge.net/projects/bbmap/) with post-alignment processing performed using SAMtools and BEDtools.

### Resequencing the genome of the SVV Delta strain

Sequencing libraries were prepared for SVV Delta and unrelated samples using the Nanopore native genomic DNA by ligation kit (SQK-LSK109) and native barcoding kit (EXP-NBD104). 1,000 ng of purified genomic DNA was used per sample and five samples were multiplexed and sequenced on a single MIN106 flowcell for 24 hours. Demultiplexing and high-accuracy basecalling were performed using Guppy v4.2.2 (-c dna_r9.4.1_450bps_hac.cfg -r—trim_strategy dna -x auto—calib_detect—trim_barcodes—barcode_kits "EXP-NBD104"). The SVV dataset (S3 Fig) was subsequently aligned against the existing SVV Delta reference genome (NC_002686.2, [6]) using MiniMap2 [47] and only aligned reads > 1,000 nt in length were retained following processing with SAMTools [48] and BBTools (https://sourceforge.net/projects/bbmap/). Filtered reads were subsequently assembled into a single circular contig using canu [51] and polished (four rounds) using racon [52] to produce a final consensus sequence (SVV Delta-EMC). SNPs and indels were identified through comparative analysis between SVV Delta and SVV Delta-EMC using base-by-base [53].

A second strategy for identifying SNPs and indels utilized the Illumina RNA-Seq dataset (S3 Fig). Here, the alignments were processed using bam-readcount (https://github.com/genome/bam-readcount) followed by processing with a custom script (variant-caller.py) available at https://github.com/DepledgeLab/vzv-2.0/tree/master/extras. Only variant SNPs and indels present at a frequency > 20% were retained for analysis.

A total of 55 SNPs were identified by nanopore sequencing and 50 by Illumina sequencing, 49 of which overlapped between datasets (S3 Fig). By contrast, nanopore sequencing identified 160 indels compared to 32 identified by Illumina sequencing, 31 of which overlapped between datasets (S3 Fig). The vast majority of nanopore-unique indels were located in homopolymers which remain problematic for this sequencing approach [54]. Thus, only SNPs and indels present in both datasets, and a single high-confidence SNP present in the Illumina dataset were considered to be accurate and their location and impact are given in S4 Table. Additionally, FASTA files containing all SNPs in CDS regions are available at: https://github.com/depledgelab/SVV-2.0.

### RNA extraction, cDNA synthesis and quantitative PCR

BS-C-1 and LLC-MK2 cells were infected with cell-associated SVV by cocultivation of uninfected and SVV-infected BS-C-1 or LLC-MK2 cells in a 8:1 ratio for 96 hrs. Cells were harvested in 1 mL Trizol, mixed with 200 μL chloroform, centrifuged for 15 min at 12,000xg at 4°C and RNA was extracted from the aqueous with the RNeasy Mini Kit (Qiagen) including on-column DNAse treatment according to manufacturer’s instructions. Similarly, RNA extraction from tissue samples was performed by homogenizing frozen tissue samples in 1 ml TRIzol using the MP Fastprep-24 (MP Biomedicals) and the RNeasy Mini Kit (Qiagen). cDNA synthesis was performed using a maximum of 5 μg of total RNA, oligo(dT) primers and Superscript IV reverse transcriptase (both Thermo Fisher). qPCR was performed in duplicate on RT- and RT+ cDNA on an ABI Taqman qPCR system using Taqman 2x Universal Master Mix or 4x Taqman Fast Advanced Master Mix (both Applied Biosystems). Relative expression was determined by normalization to β-actin using the 2^(-deltaCt) method. Primer probe sets directed to GAPDH, SVV ORF29, SVV ORF40, SVV ORF61, SVV ORF63 have been described before [23], and primer-probes sets directed against SVV VLT have been designed and validated in this study. All primer and probe sequences are given S5 Table.

### RT-PCR and Sanger sequencing analysis

PCR was performed on both RT- and RT+ cDNA samples with Amplitaq Gold DNA Polymerase (Thermo Fisher Scientific) or PfuUltra II Fusion High-fidelity DNA polymerase (Agilent) and primer pairs corresponding to the newly identified SVV transcripts (S5 Table). PCR amplification was performed by initial denaturation at 95°C for 10 min, followed by 40 cycles of denaturation (30 s, 95°C), primer annealing (50°C for 30 s) and subsequent primer extension (1–1.5 min at 72°C), and finally a single extension step of 10 min at 72°C. Adaptor-based and nested PCRs were performed with the same protocol on PCR reactions purified by Qiagen MinElute Reaction Clean up kit. For strand-specific RT-PCR, we first generated strand-specific cDNA using K20T primers (consisting of 20 thymidines and an “adaptor K” sequence) (S5 Table) and subsequently used transcript-specific forward primer and “adaptor K” specific reverse primer (PrK) (S5 Table). For RNA13.5–1, the strand-specific PCR was purified and followed by a semi-nested PCR with RNA13.5–1 specific forward and reverse primers. Amplicons were visualized on an agarose gel, excised from gel, purified using the Qiaquick Gel Extraction kit and subsequently sequenced with the BigDye v3.1 Cycle Sequencing Kit (Applied Biosystems) on an ABI Prism 3130 XL Genetic Analyzer.

### 5’ Rapid amplification of cDNA ends (RACE)

5’RACE was performed on 5 μg RNA isolated from ganglia at 13 and 21 dpi, and on 10 μg RNA isolated from lytically infected B-SC-1 cells, with the FirstChoice RLM-RACE Kit (ThermoFisher Scientific) according to manufacturer’s instructions. Finally, cDNA was generated in a total volume of 20 μL. Nested PCR reactions were performed, and cloned into pC4-TOPOvector using the TOPO-TA Cloning kit (ThermoFisher Scientific). Finally, individual colonies were picked and sequenced on the ABI Prism 3130 XL Genetic Analyzer using supplied M13 forward and reverse primers.

### RNAScope *in situ* hybridization (ISH)

ISH was performed using the RNAScope 2.5 HD Assay and probes directed to SVV VLT (core VLT exons 1–3; cat# 549461), SVV ORF63 (cat# 438091), universally expressed positive control gene ubiquitin C (UBC) and negative control bacterial transcript DapB (all from Advanced Cell Diagnostics). Staining was visualized using FastRed as a substrate, nuclei were stained with hematoxylin and slides were mounted with Ecomount (Biocare Medical). ISH was performed on normal skin and varicella skin rash of n = 2 animals (AGM 269 and 279 in [23]), with n = 2 independent experiments and 3–4 skin biopsies per tissue section. Additionally, we performed ISH on 21 DRG from n = 2 animals (RM 2207 and RM 9021, [40]), encompassing 3 or 4 sacral ganglia and 7 lumbar ganglia per animal.

## Supporting information

S1 Fig Example of strategy used to annotate the SVV transcriptome. Coverage plot denoting two major transcription units (TU) in the SVV genome. The first TU includes RNAs 18–1 and 19–1 while the second TU consists of a single RNA (20–1). To define RNAs and TUs, Nanopore dRNA-Seq (light blue) of lytically SVV-infected BS-C-1 cells was integrated with pileup data that maps the pileup of 5’ (red) and 3’ (black) ends of polyadenylated RNAs mapping to this region. Rows denoted by transcription start sites (TSS, red) and cleavage and polyadenylation sites (CPAS, black) indicate positions of putative TSS and CPAS identified using HOMER [55]. TSS and CPAS that are followed or preceded by a change in coverage are included to define transcript boundaries, indicated by asterisk. Note that within a transcription unit, putative TSS were conservatively rejected as transcript boundaries if their depth was less than 10% of the depth of the major TSS within the same transcription unit. RNA structures (gray) are inferred from these sites. Wide and thin boxes indicate canonical coding sequence (CDS) domains and untranslated regions (UTRs), respectively.
(TIF)

S2 Fig Classification of RNA15 as ncRNA. (A) Schematic representation of the 3’end of RNA15 and RNA16. The nucleotide sequence including polyadenylation site, cleavage site and U-rich DSE are indicated. The transparent box indicates the predicted CDS of RNA15, extending beyond the 3’end as defined by dRNA-seq. (B) Schematic representation of all RNA15 and RNA16 isoforms (purple boxes), the DNA sequence and the amino acids encoded by all three open reading frames with start codons (M) highlighted in green and stop codons (*) in red. The bottom row of amino acids indicates the open reading frame from which pORF15 is translated.
(TIF)

S3 Fig Sequencing and de novo assembly of the SVV Delta-EMC genome. (A) Schematic outline of sequencing and assembly approaches used to assembly the SVV Delta-EMC genome and perform comparative analysis vs. the canonical SVV Delta assembly. Venn diagrams indicate the numbers of SNPs and Indels identified by Nanopore gDNA sequencing and high-coverage Illumina RNA-Seq. (B) Schematic overview of the SVV Delta-EMC genome. SNPs and Indel differences relatively to the canonical SVV Delta genome (GenBank NC_002686.2) are highlighted by black (non-synonymous SNP), grey (synonymous SNP), red (SNP in non-coding region), and green (Indel) vertical lines. CDS impacted by non-synonymous SNPs and Indels are highlighted in black and green, respectively. Note the cross-hatched ORF52 contains both a non-synonymous SNP and an Indel.
(TIF)

S4 Fig SVV VLT is predicted to encode a homologue of VZV pVLT. (A) Minimal structural requirements to encode for each SVV pVLT variant: exons (grey boxes), introns (dashed line) and ATG (green arrow). Many other isoforms with longer 5’ UTRs can also encode for these pVLT variants. (B) Total number of annotated VLT isoforms encoding for indicated SVV pVLT variants. (C) Multiple sequence alignment by Clustal Omega of all SVV pVLT variants and VZV pVLT indicates a similar core of the protein with diversification at the N- and C-terminus. Colors are added for visual comparison. (D) Pairwise sequence alignment by EMBOSS Needle of SVV pVLT (145 aa) and VZV pVLT. Lines indicate identical amino acids (aa) and double and single points degree of structural similarity. (E) Percentage amino acid identity between SVV and VZV pVLT and VLT surrounding proteins (n = 3 upstream, n = 3 downstream) in both viruses.
(TIF)

S5 Fig Confirmation of SVV VLT expression during lytic infection of LLC-MK2 cells and *in vivo*. (A) RT-PCR confirmation of the core of SVV VLT indicates 3 major isoforms in LLC-MK2 cells. (B) RT-PCR confirmation of 4 upstream exons represented by cluster C, D, E and G (see Fig 6) in LLC-MK2 cells. (C) RT-qPCR for several lytic genes and VLT on RNA extracted from SVV-infected LLC-MK2 cells at 96 hpi (n = 2). (D) Detection of negative control DAPB RNA by in situ hybridization in consecutive sections of varicella skin lesions of an SVV-infected AGM at 9 dpi. Sections were counterstained with hematoxylin.
(TIF)

S1 Table Overview of sequence datasets. (XLSX)

S2 Table TSS and CPAS sites identified within the SVV genome. (XLSX)

S3 Table Complete SVV reannotation and coding potential. (XLSX)

S4 Table Comparison between SVV Delta-EMC and current SVV Delta GenBank reference sequence (NC_002686.2). (XLSX)

S5 Table Primers used in this study. (XLSX)

S6 Table Abundance of transcript isoforms in LLC-MK2 and CM datasets. (XLSX)
