# Supplementary figures and images for: The architecture of the simian varicella virus transcriptome
Source: PLoS Pathog. 2021 Nov 22;17(11):e1010084. doi: 10.1371/journal.ppat.1010084 (PMC8648126; doi:10.1371/journal.ppat.1010084)

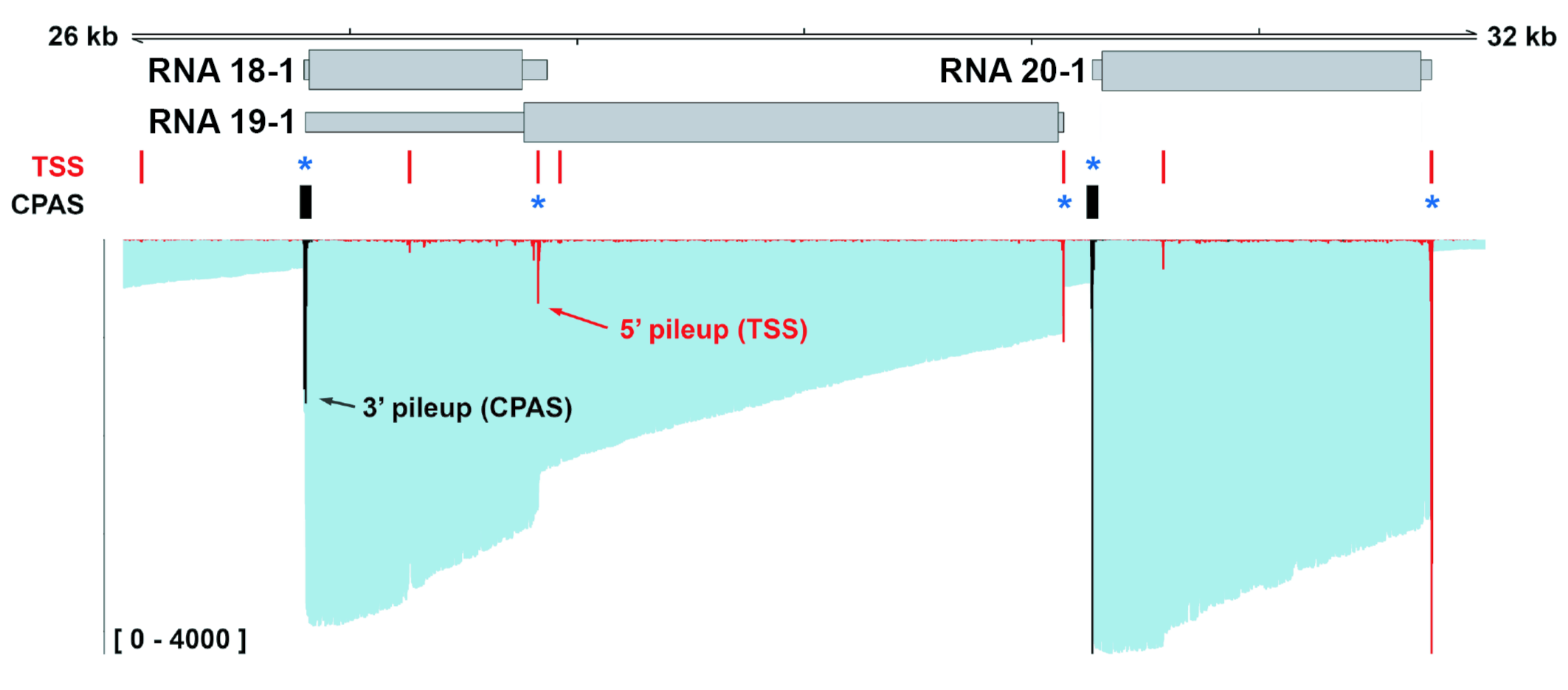

Supplement: S1 Fig — Coverage plot denoting two major transcription units (TU) in the SVV genome. The first TU includes RNAs 18–1 and 19–1 while the second TU consists of a single RNA (20–1). To define RNAs and TUs, Nanopore dRNA-Seq (light blue) of lytically SVV-infected BS-C-1 cells was integrated with pileup data that maps the pileup of 5’ (red) and 3’ (black) ends of polyadenylated RNAs mapping to this region. Rows denoted by transcription start sites (TSS, red) and cleavage and polyadenylation sites (CPAS, black) indicate positions of putative TSS and CPAS identified using HOMER [55]. TSS and CPAS that are followed or preceded by a change in coverage are included to define transcript boundaries, indicated by asterisk. Note that within a transcription unit, putative TSS were conservatively rejected as transcript boundaries if their depth was less than 10% of the depth of the major TSS within the same transcription unit. RNA structures (gray) are inferred from these sites. Wide and thin boxes indicate canonical coding sequence (CDS) domains and untranslated regions (UTRs), respectively. (TIF) [file ppat.1010084.s001.tif]

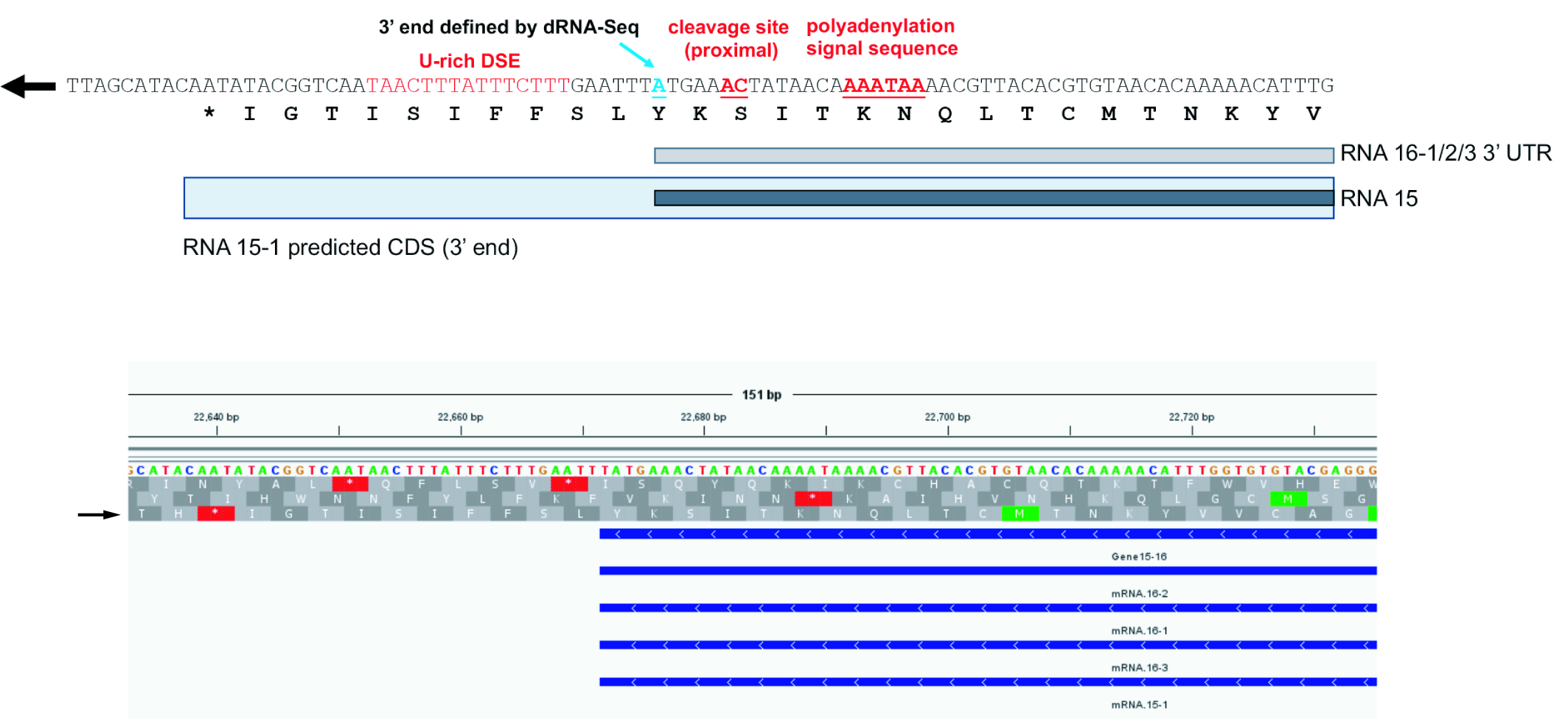

Supplement: S2 Fig — (A) Schematic representation of the 3’end of RNA15 and RNA16. The nucleotide sequence including polyadenylation site, cleavage site and U-rich DSE are indicated. The transparent box indicates the predicted CDS of RNA15, extending beyond the 3’end as defined by dRNA-seq. (B) Schematic representation of all RNA15 and RNA16 isoforms (purple boxes), the DNA sequence and the amino acids encoded by all three open reading frames with start codons (M) highlighted in green and stop codons (*) in red. The bottom row of amino acids indicates the open reading frame from which pORF15 is translated. (TIF) [file ppat.1010084.s002.tif]

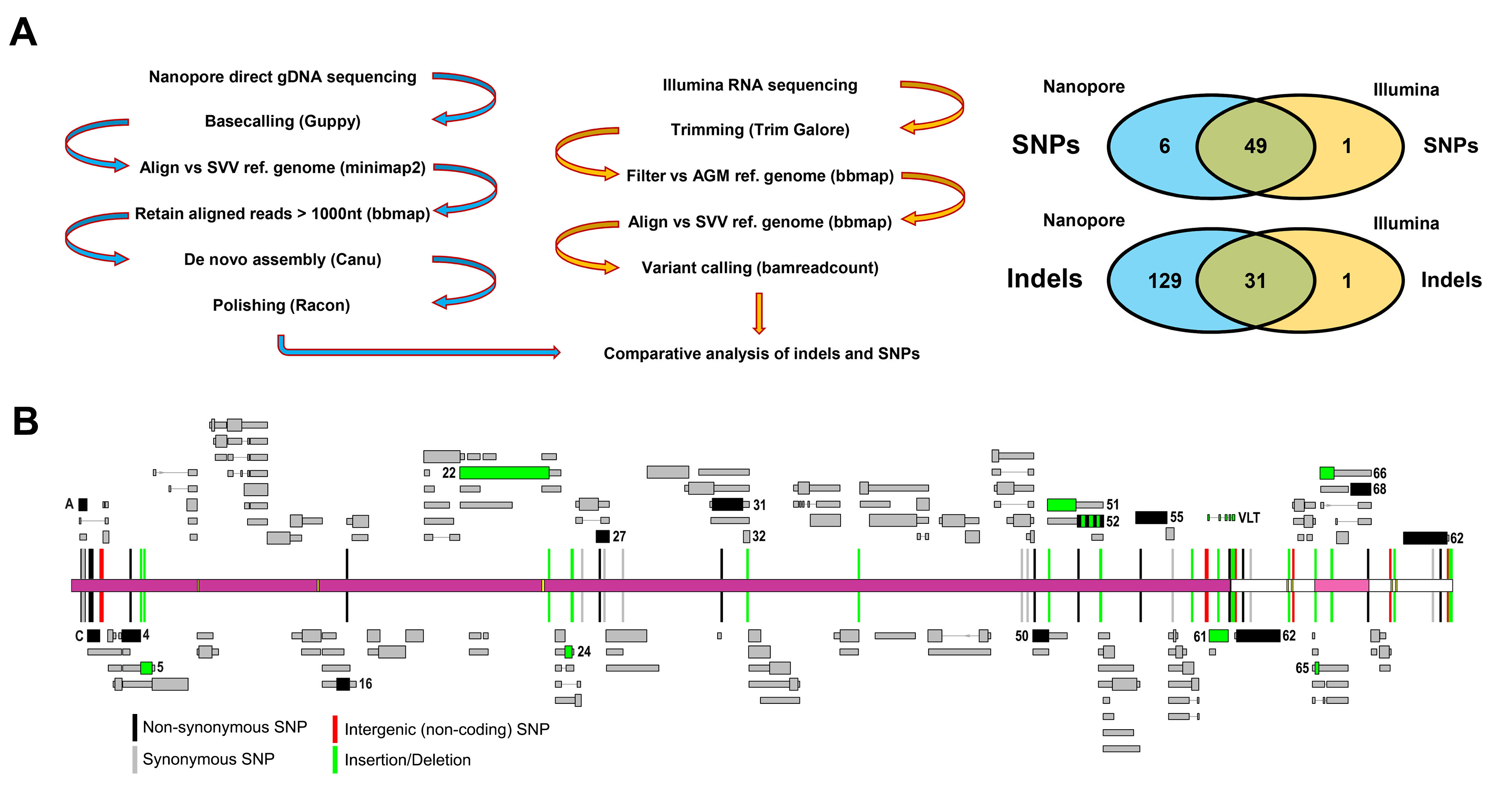

Supplement: S3 Fig — (A) Schematic outline of sequencing and assembly approaches used to assembly the SVV Delta-EMC genome and perform comparative analysis vs. the canonical SVV Delta assembly. Venn diagrams indicate the numbers of SNPs and Indels identified by Nanopore gDNA sequencing and high-coverage Illumina RNA-Seq. (B) Schematic overview of the SVV Delta-EMC genome. SNPs and Indel differences relatively to the canonical SVV Delta genome (GenBank NC_002686.2) are highlighted by black (non-synonymous SNP), grey (synonymous SNP), red (SNP in non-coding region), and green (Indel) vertical lines. CDS impacted by non-synonymous SNPs and Indels are highlighted in black and green, respectively. Note the cross-hatched ORF52 contains both a non-synonymous SNP and an Indel. (TIF) [file ppat.1010084.s003.tif]

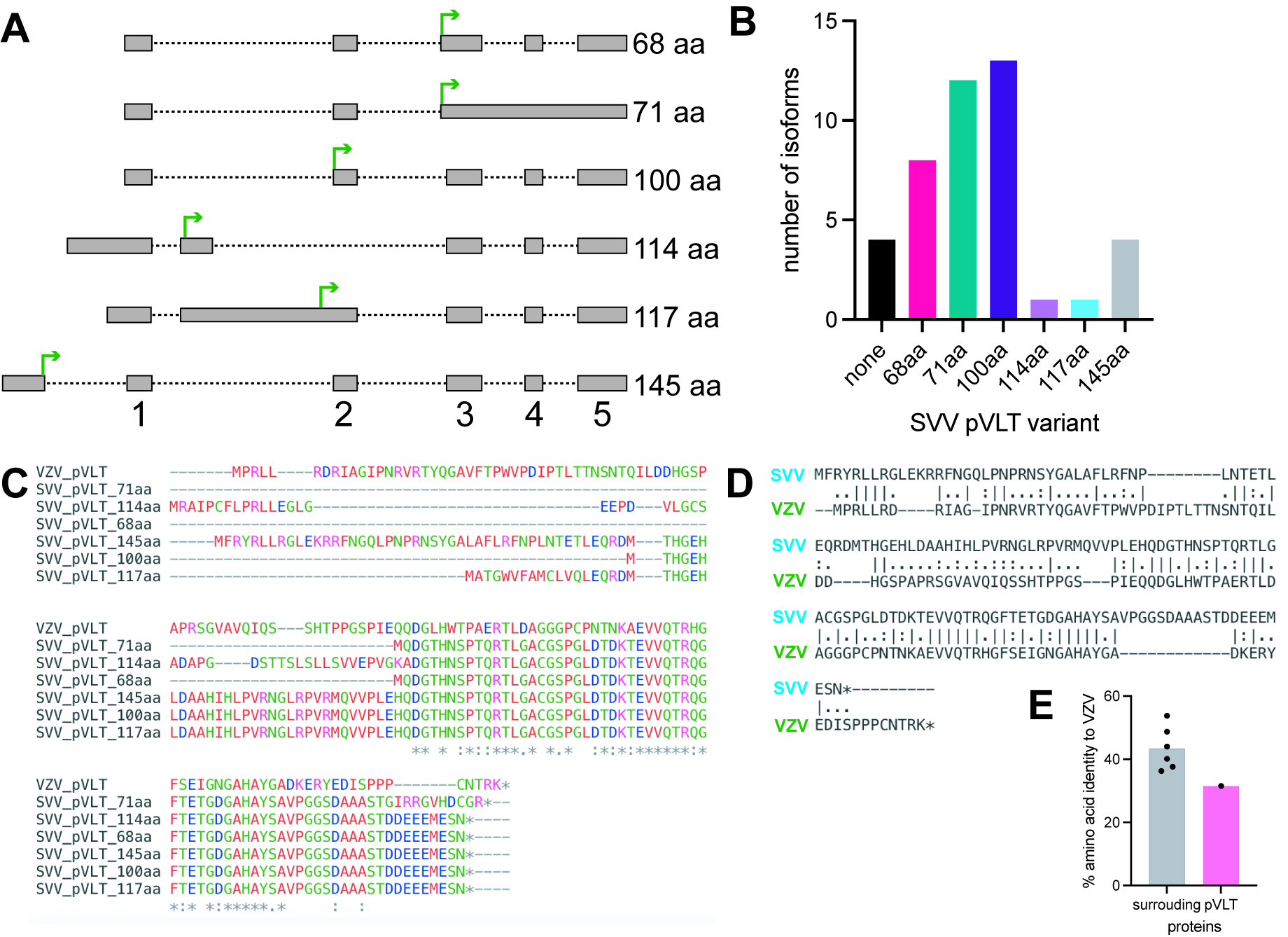

Supplement: S4 Fig — (A) Minimal structural requirements to encode for each SVV pVLT variant: exons (grey boxes), introns (dashed line) and ATG (green arrow). Many other isoforms with longer 5’ UTRs can also encode for these pVLT variants. (B) Total number of annotated VLT isoforms encoding for indicated SVV pVLT variants. (C) Multiple sequence alignment by Clustal Omega of all SVV pVLT variants and VZV pVLT indicates a similar core of the protein with diversification at the N- and C-terminus. Colors are added for visual comparison. (D) Pairwise sequence alignment by EMBOSS Needle of SVV pVLT (145 aa) and VZV pVLT. Lines indicate identical amino acids (aa) and double and single points degree of structural similarity. (E) Percentage amino acid identity between SVV and VZV pVLT and VLT surrounding proteins (n = 3 upstream, n = 3 downstream) in both viruses. (TIF) [file ppat.1010084.s004.tif]

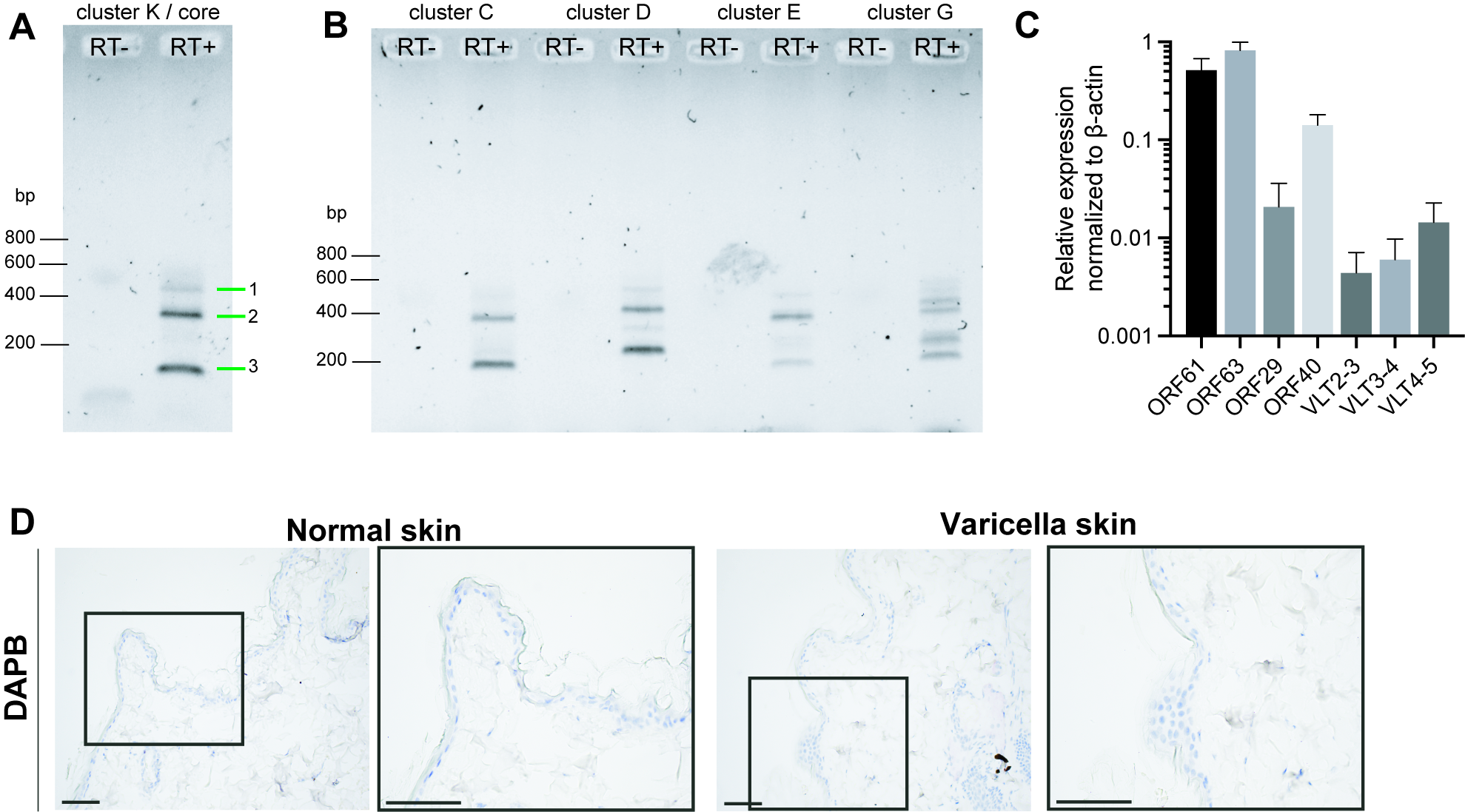

Supplement: S5 Fig — (A) RT-PCR confirmation of the core of SVV VLT indicates 3 major isoforms in LLC-MK2 cells. (B) RT-PCR confirmation of 4 upstream exons represented by cluster C, D, E and G (see Fig 6) in LLC-MK2 cells. (C) RT-qPCR for several lytic genes and VLT on RNA extracted from SVV-infected LLC-MK2 cells at 96 hpi (n = 2). (D) Detection of negative control DAPB RNA by in situ hybridization in consecutive sections of varicella skin lesions of an SVV-infected AGM at 9 dpi. Sections were counterstained with hematoxylin. (TIF) [file ppat.1010084.s005.tif]
